# Active-site arginines differentially control Cas12a DNA cleavage and specificity

**DOI:** 10.1016/j.jbc.2026.113560

**Published:** 2026-09-16

**Authors:** Saadi Rostami, Alberto M. dos Santos, Richard S. Van, Kole Long, Yihan Shao, Rakhi Rajan

**Affiliations:** 1 Department of Chemistry and Biochemistry, Price Family Foundation Institute of Structural Biology, Stephenson Life Sciences Research Center, The University of Oklahoma, Norman, Oklahoma, USA; 2 Department of Chemistry, Brandeis University, Waltham, Massachusetts, USA

**Keywords:** CRISPR-cas, protein engineering, enzyme kinetics, substrate specificity, DNA-protein interaction, Cas12a, off-target DNA cleavage, *trans* cleavage, RuvC, high-fidelity variants

## Abstract

Cas12a is a CRISPR-Cas nuclease with biochemical features that make it useful for genome editing and nucleic acid diagnostics. However, its off-target and non-specific *trans* and CRISPR RNA-independent DNA cleavages can reduce the accuracy and limit applications requiring high fidelity. Here, we analyzed the role of two conserved arginine residues, R918 and R921, found in the RuvC active site pocket of *Francisella novicida* Cas12a. Through amino acid substitutions, biochemical assays, kinetic analysis, and computational study, we establish that a positive charge at 921 is required for CRISPR RNA-dependent DNA cleavage (*cis* cleavage), whereas R918 primarily enhances cleavage efficiency. Replacing R918 with lysine or alanine eliminates *trans* activity while retaining *cis* cleavage, whereas replacing R921 with lysine eliminates *trans* activity and replacing with alanine abolishes *cis* and *trans* cleavages. Furthermore, these changes significantly decrease RNA-independent cleavage and improve mismatch discrimination during *cis* cleavage, especially at PAM-distal sites. Structural analysis shows that R918 assists in the conversion of the lid covering the RuvC active site to an alpha helical form, while R921 stabilizes the DNA in the active site. Molecular dynamics simulations reveal that while R921 is critical for supporting the positioning of scissile phosphate, R918 is essential in maintaining catalytic-site organization through lid’s conformational change as well as in positioning DNA through its role in stabilizing the active site framework. Together, our results highlight the importance of R918 and R921 in Cas12a′s activity and the potential of modifying active pocket residues to reduce unwanted DNA cleavage while increasing on-target specificity.

Clustered Regularly Interspaced Short Palindromic Repeats (CRISPR)-associated (Cas) systems are adaptive immune mechanisms found in bacteria and archaea that help them defend against invading foreign genetic materials by targeting and cleaving them. This natural mechanism has been harnessed for genome editing, with Cas proteins playing a key role in recognizing and cleaving specific DNA sequences (1, 2, 3). Among these, Cas12a has gained attention not only as a gene-editing tool but also as a diagnostic platform since it possesses additional DNA cleavage properties (4, 5, 6). Cas12a exhibits three different DNA cleavage activities: *cis* cleavage, *trans* cleavage, and CRISPR-RNA (crRNA)-independent (R-I) cleavage (7, 8, 9). During *cis* cleavage, Cas12a utilizes crRNA to find target DNA with 20-nucleotides (nt) long complementarity with the guide region of the crRNA. This target site has to be flanking a DNA motif called the protospacer adjacent motif (PAM) (10). Once PAM is recognized, the guide region of the crRNA forms a 20-nt long R-loop with the target DNA (11). After fulfilling these requirements, Cas12a cleaves both strands of the double-stranded DNA (dsDNA) using its RuvC endonuclease domain and creates a staggered end that is 4 or 5-nt long (7, 11). The cleavage occurs sequentially with the single-stranded DNA (ssDNA) of the R-loop (called the non-target strand, NTS) being cut first, followed by cleavage of the strand that base pairs with the crRNA (called the target strand, TS) (11). In *trans* cleavage, Cas12a non-specifically degrades ssDNA following *cis* cleavage when the RuvC stays in an active conformation and is accessible for binding and cleaving other ssDNAs (8, 12). Furthermore, in R-I cleavage, Cas12a degrades ssDNA into short oligos and nicks dsDNA in the absence of the crRNA, and this activity is enhanced by specific divalent metal ions such as Mn^2+^ and Co^2+^ (9, 13). The *cis* and *trans* DNA cleavage activities of Cas12a have enabled its use in applications ranging from genome modifications to nucleic acid-based biomarker detection (8).

Structurally, Cas12a consists of two lobes: the recognition (REC) lobe and the nuclease (NUC) lobe, which are bridged by a conserved arginine/lysine-rich helix known as the bridge helix (14, 15, 16, 17, 18, 19, 20) (Fig. 1*A*). DNA cleavage activity is performed by the RuvC domain in the NUC lobe. The elongated structure of the apo-Cas12a is converted into a crab-claw shaped structure after it binds to the crRNA (21). Binding of target DNA triggers structural changes in Cas12a, including shifts in the REC lobe and bridge helix (12, 16, 18, 22), expansion of the central channel for RNA-DNA hybrid formation (16, 23), displacement of the NTS into the RuvC cleft (11), and reorganization of the RuvC-lid (15), resulting in full R-loop formation. Following this, the NTS is initially cleaved, enabling the subsequent cleavage of the TS (7, 14, 16, 24). In the initial stages of the conformational cascade necessary for Cas12a′s DNA cleavage, the RuvC active pocket is occluded by an unstructured loop, referred to as the "lid". In the later stages, when R-loop reaches 16-nt of base pairing, the conformational change in the lid region induces the formation of an α-helix, thereby exposing the RuvC active site for DNA entry (25). Previous studies have demonstrated that the α-helical transition of the lid plays a critical role not only in RuvC activation but also in guiding the TS toward the active site for cleavage (24, 25, 26).

**Figure 1:**
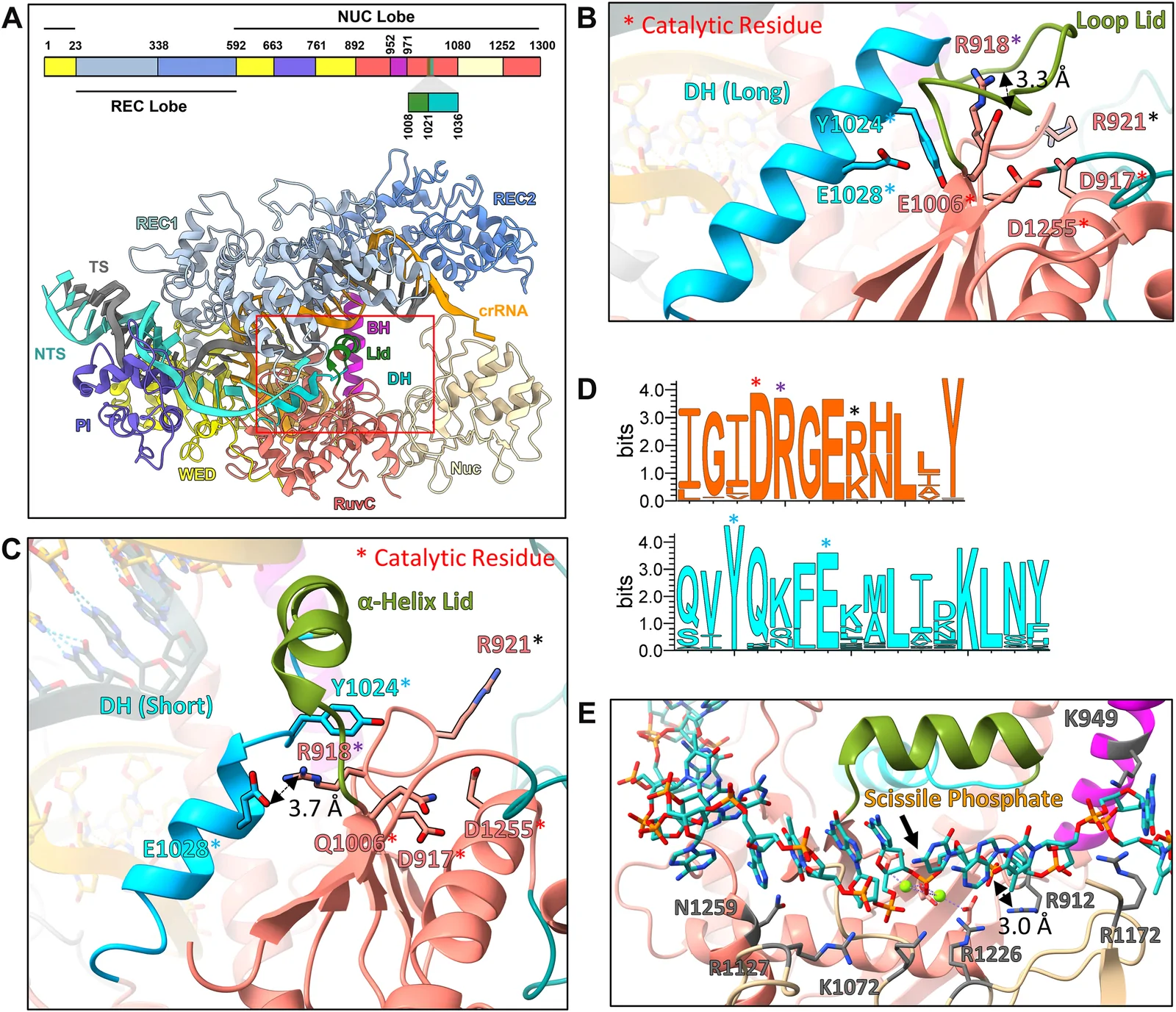
**Structure and sequence analyses highlight how RuvC-lid’s conformational changes and conserved residues in the active pocket regulate active-site accessibility and NTS-DNA interaction in Cas12a.***A*, domain representation of FnoCas12a (PDB ID: 6GTG). The protein is composed of the recognition (REC) lobe, including REC1 (recognition domain 1) and REC2 (recognition domain 2), and the nuclease (NUC) lobe, containing the WED (wedge) domain, PI (PAM-interacting) domain, BH (bridge helix), RuvC nuclease domain, Lid (RuvC-lid region), DH (downstream helix), and Nuc domain. The crRNA is shown in orange, the target strand (TS) in *gray*, and the non-target strand (NTS) in turquoise. The boxed region highlights the active-pocket region analyzed in this study. *B*, the RuvC active site of FnoCas12a (PDB ID: 6I1K) is occluded by loop form of the RuvC-lid (*green*). Residue R918 restrains E1006, one of the catalytic residues. Both of these features stabilize RuvC’s inactive conformation. Downstream helix is in *cyan* and rest of RuvC domain is in salmon. *C*, the RuvC-lid (*green*) is folded into an alpha helix, rendering the active site accessible for DNA (PDB ID: 6GTG). R918 releases E1006 and instead interacts with E1028 in the downstream helix (*cyan*). In addition, the shortening of the downstream helix may facilitate the RuvC-lid’s conformational transition. In this structure, the catalytic residue E1006 is substituted with glutamine (Q) to inactivate DNA cleavage during structure determination. RuvC is in salmon. *D*, sequence conservation analysis of the RuvC domain across 91 different Cas12a orthologs including (*Francisella novicida*, *Acidaminococcus*, Lachnospiraceae *bacterium*, and *Moraxella bovoculi*) shows that R918, R921, Y1024, and E1028 are conserved. Only regions corresponding to RuvC (*salmon*) and downstream helix (*cyan*) are shown. *E*, the path of NTS-DNA (*turquoise*) in the active pocket of AsCas12a (PDB ID: 8SFO) is shown with the RuvC-lid (*green*) in its α-helical conformation. The chain of residues that binds NTS-DNA is highlighted and R912 (the counterpart of R921 in FnoCas12a) is shown and it interacts with the phosphate adjacent to the scissile phosphate in NTS-DNA. *Gray* residues indicate residues that interact with the NTS-DNA.

Compared to Cas9 that is widely used for gene editing and gene therapy (1, 27, 28), Cas12a offers several advantages, including the ability to create staggered dsDNA breaks that enhance homologous recombination and the potential for multiplexed gene editing due to the inherent capacity of Cas12a to process and create functional crRNAs without requiring other accessory proteins (29). In addition, studies have shown that Cas12a exhibits lower off-target effects than Cas9, making it a more precise tool for gene editing (30, 31, 32). Despite these, challenges remain in utilizing Cas12a for genome editing, particularly due to its *trans* cleavage, which can negatively impact editing outcomes (33). One significant concern is that *trans* activity can degrade ssDNA donor templates used for homology-directed repair (HDR), thereby reducing the efficiency of precise genome modifications (33, 34).

To mitigate nonspecific and off-target DNA cleavages of Cas12a, we focused on two conserved arginine residues located in the RuvC active site pocket of *Francisella novicida* Cas12a (FnoCas12a), R918 and R921, that can potentially play a role in binding to substrates and/or stabilizing the folding of the RuvC lid, thereby influencing the cleavage activities. We demonstrate that replacing R918 with either alanine or lysine reduces non-specific and off-target activities while preserving on-target cleavage. In contrast, substitutions at R921 exert a stronger effect on catalytic function, with lysine retaining partial on-target activity and alanine eliminating it. Molecular dynamics simulations show that R918 primarily supports RuvC-lid’s loop-to-helix conversion and active-site organization, thereby indirectly promoting DNA coordination, whereas R921 directly mediates DNA positioning within the active site. As such, we engineer variants of FnoCas12a with no *trans* activity, reduced R-I activity, and improved RNA–DNA mismatch discrimination. Our results highlight rational engineering strategies that fine-tune protein–DNA contacts and active site conformations to improve the precision of Cas12a and potentially other Cas enzymes.

## Results

### Arginines near the RuvC active-site pocket contribute to attaining the catalytic state

To investigate the potential role of arginine residues located near the active pocket of FnoCas12a′s RuvC, we compared two structures of Cas12a-crRNA-DNA complex, PDB ID: 6I1K (11), in which the RuvC-lid region adopts a flexible loop conformation that blocks the active pocket, and PDB ID: 6GTG (24), a transition state structure, in which the RuvC-lid folds into an α-helix, leaving the pocket accessible for ssDNA binding. In the loop conformation, R918 forms a polar interaction with E1006, one of the catalytic residues, thereby restraining it from achieving the orientation needed for promoting the catalytic reaction (Fig. 1*B*). Upon folding of the RuvC-lid into an α-helix, R918 disengages from E1006 and shifts to interact with E1028 in the middle of the downstream helix (Fig. 1*C*). This transition is accompanied by shortening of the downstream helix due to some helical turns of this helix unwinding and contributing to the loop between the downstream helix and the α-helical RuvC-lid, which may provide the flexibility needed for the folding of the RuvC-lid (Fig. 1, *B* and *C*). R918 is highly conserved among Cas12a orthologs (Fig. 1*D*; Fig. S1), and its counterpart (R909) in *Acidaminococcus* sp. Cas12a (AsCas12a) exhibits the same interaction pattern during RuvC-lid’s conformational changes (25) (Fig. S2; Movie S1). Structural snapshots of AsCas12a that capture a series of steps during the R-loop formation further reveal an intermediate interaction of R909 (the counterpart of FnoCas12a′s R918) with Y1012 (the counterpart of FnoCas12a′s Y1024) in the downstream helix (Fig. S2, *B*–*D*) (25), followed by the formation of a hydrogen bond with E1016 (the counterpart of FnoCas12a E1028) of the downstream helix (Fig. S2, *E*–*H*) (25), supporting a stepwise process during the formation of and stabilization of the RuvC-lid helix in different orthologs. The interaction of AsCas12a R909 with E1016 appears to be maintained through cleavage of both DNA strands and returning to its initial interaction with E993 (the counterpart of E1006 in AsCas12a) after the structure resets following both strand cleavages (Fig. S2, *E*–*I*) (25). This resetting is accompanied by a transition of the RuvC-lid from α-helical conformation back to loop state (Fig. S2, *H* and *I*). These observations suggest that the differential conformations of R918 contribute to regulating catalytic activity. Because RuvC-lid’s dynamic motions may provide continued access to the RuvC catalytic pocket for the progressive *trans* cleavage activity, we hypothesized that substitution of this residue may severely impact *trans* activity, compared to *cis* cleavage, which is a single-turnover event at a single on-target site (8, 35).

In contrast, the structures of FnoCas12a (PDB IDs: 6I1K (11) and 6GTG (24)) do not indicate that R921 drives conformational changes in the RuvC-lid region but point to possible roles in placing the DNA in the RuvC active site pocket. Although full NTS DNA is absent in available FnoCas12a structures, the full NTS DNA in AsCas12a active pocket is captured in the full R-loop state (PDB ID: 8SFO (25)) (Fig. 1*E*). R912 in AsCas12a, the equivalent of R921 in FnoCas12a, is located within the NTS-DNA channel and forms part of a cluster of positively charged residues lining this binding pocket. Its side chain is positioned near the phosphate group adjacent to the scissile phosphate whose ester bond is cleaved during NTS-DNA cleavage (Fig. 1*E*). This unique positioning suggests that R912 (and R921 in FnoCas12a) not only stabilizes the NTS strand but may also influence cleavage efficiency through its proximity to the catalytic center. Moreover, because phosphate-mediated catalysis has been proposed as an alternative reaction pathway to the histidine-mediated mechanism in RuvC-like nucleases (such as SpyCas9 RuvC (36, 37)), the role of R921 may extend beyond simple DNA stabilization. To investigate whether the NTS-binding channel is conserved in FnoCas12a, we superimposed AsCas12a (PDB ID: 8SFO) and FnoCas12a (PDB ID: 6GTG) and generated a structural model to predict the binding pose of the NTS DNA through FnoCas12a. Following energy minimization, the model suggests that residues corresponding to NTS-interacting residues in AsCas12a form a similar channel in FnoCas12a (Fig. S3). Based on these observations, we hypothesized that substituting R921 could fine-tune Cas12a′s DNA cleavage activity by modulating substrate positioning and catalytic chemistry within the RuvC active-site pocket.

Arginine is important in protein-nucleic acid interactions because of the multivalent coordination provided by the bidentate hydrogen bonds and electrostatic interactions with the sugar-phosphate backbone or the base of a nucleotide and the stacking interactions that are possible through the planar guanidinium group of the arginine side chain. In comparison, lysine preserves positive charge that can interact with the nucleic acid but cannot fully reproduce interaction geometry provided by an arginine (38, 39). To test the effect of the two selected arginines in RNA-guided and non-specific DNA cleavages of FnoCas12a, we assessed several substitutions: *(i)* lysine substitutions to assess the role of positive charge (R918K, FnoCas12a^R918K^ and R921K, FnoCas12a^R921K^), *(ii)* alanine substitutions to remove both positive charge and side chain interactions (R918A, FnoCas12a^R918A^ and R921A, FnoCas12a^R921A^), and *(iii)* a double lysine substitution (R918K/R921K, FnoCas12a^R918K/R921K^). The wild type (FnoCas12a^WT^) and these variants were recombinantly expressed and purified (Fig. S4) (17, 40) to test the effects of reduced and no positive charge at these positions and to assess if one of the arginine(s) have a more significant role to play in catalysis compared to the other.

### R918 fine-tunes *cis* DNA cleavage efficiency, while R921 is catalytically essential

Since Cas12a variants developed for improved fidelity in genome application should retain sufficient crRNA-dependent DNA cleavage (*i.e.*, the *cis* cleavage), we tested the efficiencies of the different variants using previously reported plasmid based *cis* cleavage assays (17, 40). A pUC19-based plasmid that holds a 24 nt-long complementary region with the 24 nt of the crRNA’s guide (*i.e.*, a matched, MCH-DNA) and flanking the 5′-TTTA-3′ PAM for FnoCas12a was used as the substrate. A time course assay covering times from 15 s to 1 h was performed to track the formation and disappearance of the nicked intermediate and buildup of the linear product by the variants and FnoCas12a^WT^. FnoCas12a^R918K^ shows a slower but similar rise-and-decay pattern for the nicked intermediate with the peak of 33 ± 10% at 30 s (with 24 ± 24% linear product and 43 ± 26% supercoiled substrate) compared to the peak of 28 ± 19% for FnoCas12a^WT^ at 15 s (with 59 ± 33% linear product and 14 ± 14% supercoiled substrate; Table S1). Despite this delay, FnoCas12a^R918K^ ultimately achieves near-complete linear DNA formation (96 ± 5%) after 60 min, whereas FnoCas12a^WT^ reached full linearization within 2.5 min, indicating comparable overall extent of linearization but with slower kinetics for FnoCas12a^R918K^ (Fig. 2, *A*, *B* and *G*; Fig. S5; Table S1). To calculate kinetics, band intensities corresponding to supercoiled precursor (P), nicked (N), and linear (L) DNA were quantified and normalized (Equations (1), (2), (3)). The fraction of linear product was plotted over time and fitted to a single-exponential rise model (Equation 6) to extract the observed rate constant (*k*_*obs*_) and active fraction (A). Matched plasmid DNA linearization for FnoCas12a^WT^ has a rate constant of 3.24 ± 0.13 min^−1^, while the corresponding value for FnoCas12a^R918K^ of 0.49 ± 0.43 min^−1^, showing a 6.6-fold reduction in *cis*-cleavage efficiency (Fig. 2*G*; Fig. S5; Table S2).

**Figure 2:**
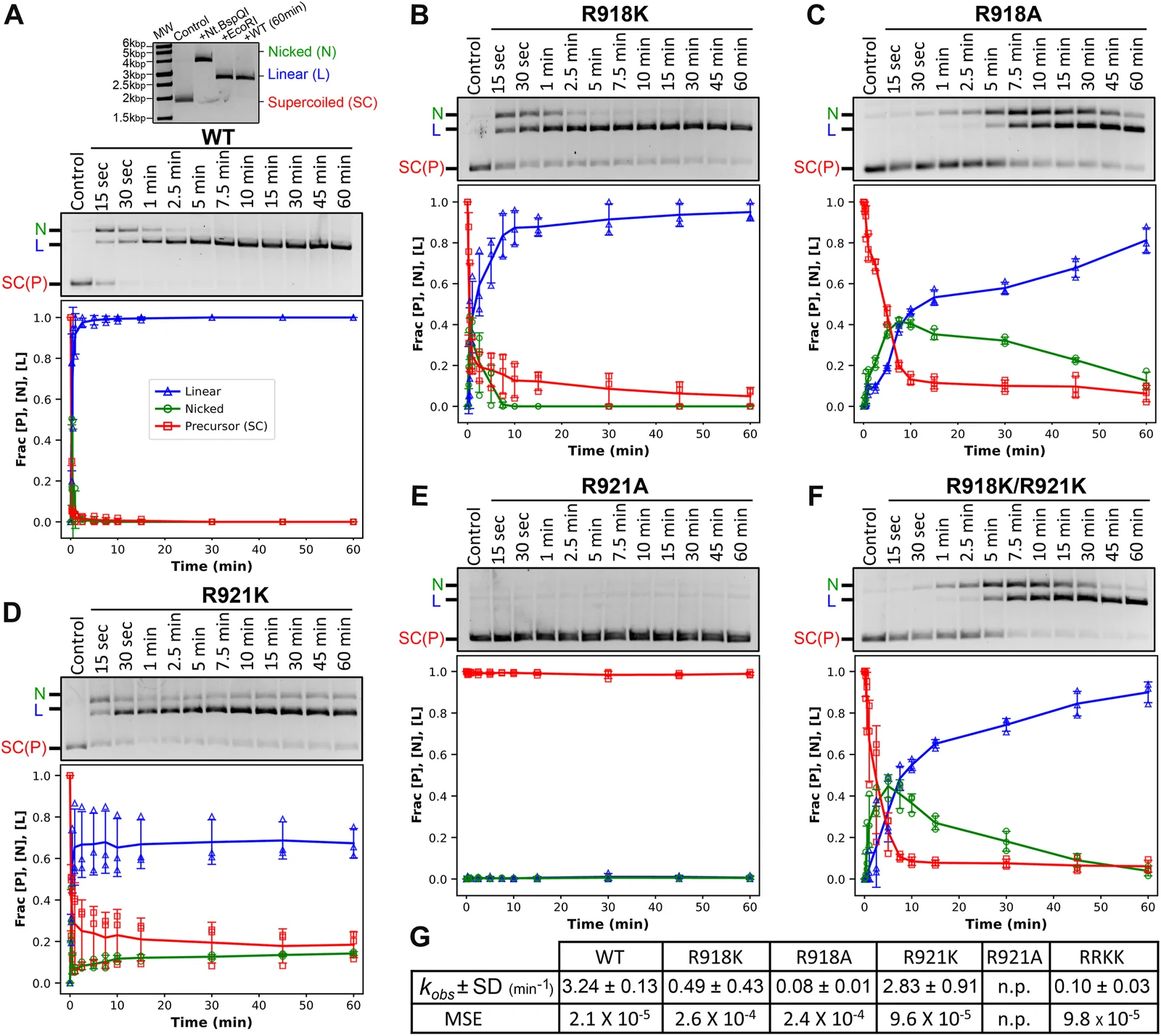
***Cis* cleavage of matched plasmid by FnoCas12a^WT^ and variants.** Representative gels showing *cis* cleavage of MCH plasmid substrate containing a 5′-TTTA-3′ PAM and 20-nt region that is complementary to the guide region of crRNA by FnoCas12a^WT^ (*A*), FnoCas12a^R918K^ (*B*), FnoCas12a^R918A^ (*C*), FnoCas12a^R921K^ (*D*), FnoCas12a^R921A^ (*E*) and FnoCas12a^R918K/R921K^^, RRKK^ (*F*) variants. In *panel A*, the *upper* reference gel shows the migration positions of different forms of MCH, including supercoiled plasmid substrate in the control lane, nicked plasmid generated by Nt.BspQI (one site in MCH plasmid), linearized plasmid generated by EcoRI (one site in MCH plasmid), and FnoCas12a^WT^-cleaved product after 60 min. A 1 kbp dsDNA ladder was used as the molecular weight (MW) marker. Individual data points represent the three independent replicates at each time point, and error bars indicate mean ± SD. The different DNA forms are labeled as N (nicked), L (linearized), and SC (supercoiled or Precursor; P). Quantification of SC, N, and L over time was plotted. *G*, the observed rate constant (k_obs_) and SD from three replications for each variant are indicated. The k_obs_ (min^−1^) of FnoCas12a^R921A^ is not provided (n.p.) because MATLAB could not reliably determine the value. Control lane contains MCH plasmid substrate incubated in reaction buffer without enzyme for 60 min.

Comparing the results with FnoCas12a^R918A^, the maximum accumulation of nicked intermediate is 42 ± 2% at 7.5 min and a maximum linearization of 81 ± 7% after 60 min. FnoCas12a^R918A^ has a 13 ± 3% accumulation of nicked intermediate after 1 h showing the inability of the variant to completely linearize the substrate after 1 h (Fig. 2*C*; Table S1). The rate constant for DNA linearization by FnoCas12a^R918A^ is 0.08 ± 0.01 min^−1^, which represents a 40.5-fold reduction compared to FnoCas12a^WT^ (Fig. 2, *A*, *C* and *G*; Fig. S5; Table S2). These results show that both FnoCas12a^R918K^ and FnoCas12a^R918A^ are capable of linearizing DNA to a maximum of 96% and 81%, respectively, after 1 h, even though it takes longer time for the variants to reach the highest activity level.

For R921, the FnoCas12a^R921K^ variant displays a similar rise-and-decay pattern for the nicked intermediate with a maximum of 32 ± 12% nicked intermediate within 15 s. FnoCas12a^R921K^ achieves 67 ± 16% linearization at 2.5 min with no further increase throughout the 60-min endpoint, with 14 ± 2% nicked intermediate and 18 ± 7% of supercoiled DNA substrate remaining at the endpoint (Fig. 2*D*; Table S1). Interestingly, the observed rate constant for FnoCas12a^R921K^ is 2.83 ± 0.91 min^−1^, which is only a 1.1-fold reduction compared to the linearization rate of FnoCas12a^WT^ (Fig. 2*G*; Fig. S5; Table S2). This indicates that R921K substitution traps at least part of the Cas12a-nucleic acid complex in a non-productive conformation despite its faster DNA cleavage compared to the other variants. The FnoCas12a^R921A^ variant has no detectable *cis* cleavage. Specifically, it is unable to either nick or linearize the DNA substrate. After 60 min of incubation, the amount of supercoiled matched plasmid remaining is comparable to the control sample without any enzyme, indicating a complete lack of *cis* cleavage by this variant (Fig. 2*E*; Table S1).

Following our analysis of single lysine substitutions for R918 and R921, we prepared a variant with a double lysine substitution (FnoCas12a^R918K/R921K^) to analyze the effect on *cis* DNA cleavage. FnoCas12a^R918K/R921K^ variant accumulates a maximum of 45 ± 5% nicked intermediate at 5 min and reaches 90 ± 5% linearization at 60 min (Fig. 2*F*; Table S1). The rate constant for DNA linearization by FnoCas12a^R918K/R921K^ is 0.10 ± 0.03 min^−1^. These results show that, compared to FnoCas12a^WT^, the cumulative effect of double lysine substitutions in FnoCas12a^R918K/R921K^ results in a 32.4-fold reduction in DNA linearization, while the single lysine substitutions have only six- or one-fold reduction (Fig. 2*G*; Fig. S5; Table S2). An interesting observation is that while FnoCas12a^R921K^ is faster with a conformational trapping (see the immediate achievement of the flat linearization line in Fig. 2*D*), such trapping is absent in FnoCas12a^R918K/R921K^ since it has a steady and more complete linearization, despite the much slower pace of the reaction (Fig. 2*F*). The current structural information of Cas12a active site does not provide molecular reasons for this behavior. Nevertheless, our results show that the enriched positive charge from two arginine(s) is critical, with significant decrease in *cis* cleavage efficiency due to the reduction in positive charge from a lysine substitution at either position. Additionally, since FnoCas12a^R918A^ is active, albeit at a slower rate, and FnoCas12a^R921A^ is inactive, R918 may play a supporting role in *cis* cleavage to increase the cleavage efficiency, while R921 is important for catalysis by positioning the scissile phosphate for the nucleophilic reaction.

### Arginines in the RuvC active pocket of Cas12a enhance DNA mismatch tolerance

Positive charge in the RuvC active site can potentially enhance binding and cleavage of off-target DNA. To assess DNA mismatch discrimination of FnoCas12a variants, we performed a plasmid cleavage assay using a series of plasmids holding a single mismatch at each of the 20 nucleotide positions that base pairs with the crRNA guide region (Fig. 3*A*). We preincubated Cas12a with crRNA at 37 °C for 10 min before adding the substrate and used only one time point, 60 min, for the mismatch assays. The results were compared to that from assays with a matched (MCH) DNA substrate. Since off-target cleavage is manifested by DNA linearization, and not by DNA nicking, we analyzed the DNA linearization potential of the wild type and variant proteins. Substrate discrimination was evaluated using two metrics: (i) R_1_: within-protein discrimination was quantified as the ratio of MCH linearization to MM linearization for each enzyme. A value of one indicates no discrimination between MCH and MM substrates; a value >1 indicates increased discrimination, with larger values reflecting stronger preferential linearization of MCH over MM; and a value between 0 and 1 indicates greater linearization of the MM substrate than the MCH substrate. (ii) R_2_: variant-to-wild-type discrimination was quantified by comparing each variant’s discrimination ratio for a particular mismatch position with that of FnoCas12a^WT^. The value of 1 indicates no change relative to the wild type; a value >1 indicates improved MM discrimination compared to FnoCas12a^WT^; and a value between 0 and 1 indicates reduced MM discrimination compared to FnoCas12a^WT^ for a specific MM DNA.

**Figure 3:**
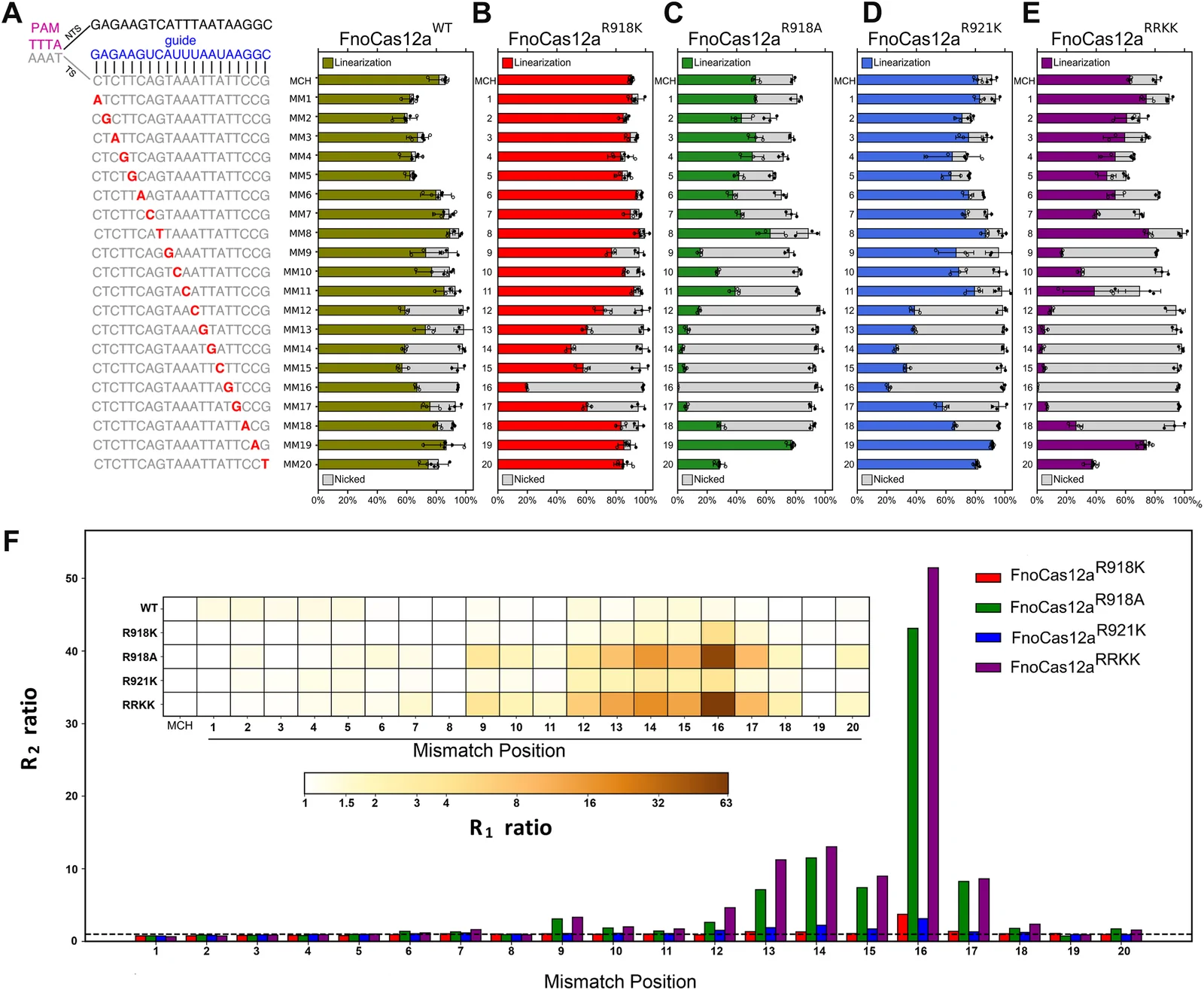
**DNA mismatch discrimination analysis for FnoCas12a^WT^ and variants during *cis* cleavage with plasmid substrates.***A*, Bar graphs show cleavage by FnoCas12a^WT^ after 1 h of incubation, where colored bars represent linearized product and *gray* stacked bars represent nicked product; together, the stacked bars represent total cleavage. Each data point shown in circles (open for linear and solid for nicked) represents one independent replicate, and error bars indicate SD from three replicates. Corresponding cleavage profiles are shown for FnoCas12a^R918K^ (*B*), FnoCas12a^R918A^ (*C*), FnoCas12a^R921K^ (*D*), and FnoCas12a^R918K/R921K^ (RRKK) (*E*). *F*, quantification of DNA mismatch discrimination potential of the variants. The heat map represents within-protein mismatch discrimination (R_1_ ratio), where darker color indicates higher efficiency for linearizing MCH than MM substrate. The bar graph shows the R_2_ ratios, calculated by dividing the MCH/MM cleavage ratio (R_1_) of each variant by that of FnoCas12a^WT^ for each MM position. Both R_1_ and R_2_ ratios show that discrimination is present for mismatches in positions 12 to 17, with the 16th position having the highest difference between FnoCas12a^WT^ and the variants. The black dashed line indicates the MM discrimination by FnoCas12a^WT^ over MCH at each position.

In general, FnoCas12a^WT^ cleaves most MM substrates as efficiently as the MCH DNA substrate (linearization ranging from 57% to 89%; Fig. 3*A*; Table S3). The within-protein discrimination (R_1_) values are closer to 1 (Ranging from 0.9 to 1.4) for most MM positions in FnoCas12a^WT^ (Fig. 3*F*; Table S4*A*), indicating low mismatch discrimination.

The FnoCas12a^R918K^ variant exhibits mismatch discrimination in positions 12 to 17 (R_1_ from 1.3 to 4.6), with the most significant being MM16 with a R_1_ ratio of 4.6 and 20 ± 0% DNA linearization (Fig. 3, *B* and *F*; Table S3; Table S4*A*). Comparing with FnoCas12a^WT^ that has 67 ± 2% linearization of MM16, FnoCas12a^R918K^ shows enhanced discrimination of MM16 (Table S3). Additionally, the R_2_ value of 3.7 indicates its ability to discriminate MM16 better than FnoCas12a^WT^ (Fig. 3*F*; Table S4*B*). Since the R_2_ ratios for all other MM positions are close to 1, FnoCas12a^R918K^ maintains superior mismatch discrimination mainly for MM16 (Table S4*B*). Interestingly, the 16th position of the RNA-DNA hybrid is a checkpoint for activation for DNA cleavage in Cas12a (25) and R918 may play a role in maintaining the checkpoint conformation.

The FnoCas12a^R918A^ variant has a severe deficiency in linearizing mismatch-containing DNA, with almost complete inhibition of linearization at positions MM13-MM17 (1–7%, Fig. 3*C*; Table S3). With a 53 ± 3% MCH linearization, the R_1_ values for FnoCas12a^R918A^ are higher for positions MM13-MM17 (ranging from 8.0 to 53.0), indicating that this variant can cleave MCH at a better efficiency compared to the MM positions (Fig. 3*F*; Table S3; Table S4*A*). Comparing this variant with FnoCas12a^WT^ shows that FnoCas12a^R918A^ has a substantial improvement in discriminating MM13-MM17 (R_2_ values ranging from 7.1 to 43.1, Fig. 3*F*; Table S4*B*). The greatest enhancement is for MM16, where the R_2_ ratio is 43.1, with only 1 ± 0% linearization of MM16 by FnoCas12a^R918A^ and a 67 ± 2% linearization by FnoCas12a^WT^ (Table S3; Table S4*B*). Beyond MM13-17, enhanced mismatch discrimination is also present for MM9 (R_1_: 3.5 and R_2_: 3.1) and MM12 (R_1_: 3.6 and R_2_: 2.6) (Fig. 3*F*; Table S4). Linearization of MM9 and MM12 by FnoCas12a^R918A^ reduces to 15 ± 2% for both, while FnoCas12a^WT^ has a linearization of 73 ± 10% and 59 ± 3%, respectively, for the same two positions (Fig. 3, *A* and *C*; Table S3). In comparison, the FnoCas12a^R918K^ variant linearizes MM9 and MM12 to 77 ± 3% and 72 ± 5%, respectively, (Table S3). The observed differences between lysine and alanine substitutions highlight the critical role of a positive charge at position 918 to enhance the cleavage of both MCH and MM substrates, with an increased need for positive charge to linearize MM13-17 positions.

The FnoCas12a^R921K^ variant results in a modest improvement in mismatch discrimination, with R_1_ values ranging from 1.4 to 3.8 and DNA linearization ranging from 21 to 58% for positions MM12-MM17 (Fig. 3*D*; Table S3; Table S4*A*). The corresponding R_2_ values range from 1.3 to 3.1. Similar to FnoCas12a^R918K^, MM16 shows the highest R_1_ and R_2_ ratios in FnoCas12a^R921K^, with values of 3.8 and 3.1, respectively (Fig. 3, *B* and *D*; Table S3; Table S4*A*). However, these values remain lower than those observed for FnoCas12a^R918A^ at the same position. Since most R_1_ and R_2_ values are close to 2, the overall mismatch discrimination potential of FnoCas12a^R921K^ remains relatively low across the different mismatch positions. Since FnoCas12a^R921A^ exhibited no detectable matched *cis* DNA cleavage, it was excluded from mismatch cleavage analyses.

The double substituent, FnoCas12a^R918K/R921K^, shows strong enhancement in mismatch discrimination across MM12-17 with an R_1_ value ranging from 6.5 to 63, with the highest discrimination for MM16 and linearization ranging from 1% for MM16 to 10% for MM12 (Fig. 3*F*; Table S3; Table S4*A*). The R_2_ value, that shows the ability of the variant to discriminate compared to FnoCas12a^WT^, ranges from 4.6 for MM12 to 51.4 for MM16 for the MM12-MM17 region (Table S4*B*). The R_2_ value of 51.4 for FnoCas12a^R918K/R921K^-MM16 is the highest improvement among all the variants tested in this study. FnoCas12a^R918K/R921K^ discriminates MM12 the best compared to all other variants tested in this study (R_1_ of 6.5, R_2_ of 4.6, with a linearization of only 10 ± 2% compared to 59 ± 3% by FnoCas12a^WT^ (Table S3; Table S4). Notably, FnoCas12a^R918K/R921K^ exceeds the expected combined effects of the individual R918K and R921K substitutions and shows superior discrimination for mismatches at positions 12 to 17 of the R-loop compared to each single variant. Comparing all the variants used in this study, FnoCas12a^R918K/R921K^ has consistently the highest R_2_ value for all the positions covering 12 to 17 of the R-loop (Table S4).

Analyzing the DNA cleavage patterns for the different variants and different mismatch positions show important mechanistic features. Almost all the variants are able to nick all the different substrates efficiently (Fig. 3, *B*–*E*). For FnoCas12a^WT^, total cleavage (*i.e.*, nicked + linear) is above 60% for all substrates (Table S3). Although nicked intermediate accumulates at 1 h across most mismatch positions, MM12-17 are the most affected, showing 17 to 39% accumulation of nicked DNA in FnoCas12a^WT^. In FnoCas12a^R918K^, this positional effect becomes more pronounced, with MM12-17 exhibiting a higher accumulation of nicked intermediate with a range of 26% to 79% (Fig. 3*B*; Table S3). The FnoCas12a^R918A^ variant displays an even more elevated response for MM12-MM17 reaching 82 to 95% of nicked intermediate, exceeding both FnoCas12a^WT^ and FnoCas12a^R918K^ (Table S3). Moreover, additional positions such as MM9-MM11 also show substantial nicked accumulation in FnoCas12a^R918A^ (42–60%), whereas the same region accumulates only 7 to 15% in FnoCas12a^WT^ and 4% to 19% in FnoCas12a^R918K^ (Table S3). The results for FnoCas12a^R921K^ closely mirror those of FnoCas12a^R918K^, with the strongest nicked accumulation for MM12-MM17, ranging from 38 to 78% (Table S3). The double substituent, FnoCas12a^R918K/R921K^, shows a similar effect with MM12-MM17 positions accumulating 84 to 95% nicked intermediate. Additionally, FnoCas12a^R918K/R921K^ displays substantial nicked accumulation for positions MM9 and MM10 (64% and 55%, respectively), exceeding the levels observed in other variants (Table S3). These differences in nicked intermediate accumulation further highlight a strong positional effect on mismatch cleavage efficiency. While the most impact is in the amount of linearization for positions MM12-MM17 in several of the variants, there is efficient production of nicked DNA in these cases. Thus, for these positions and variants, the discriminatory effect is most evident in the second-strand cleavage step where additional conformational changes are needed to bring the TS to the RuvC active site in a single-stranded form. It is interesting to note that position MM20 has reduced total cleavage in both FnoCas12a^R918A^ and FnoCas12a^R918K/R921K^ with only 28 ± 3% and 38 ± 3% total cleavage for FnoCas12a^R918A^ and FnoCas12a^R918K/R921K^, respectively (Fig. 3, *C* and *E*; Table S3). The total cleavage for FnoCas12a^WT^ is 81 ± 5% for MM20 (Table S3). These may point to additional roles that the arginines are playing in supporting the PAM-distal nt-mismatches during full R-loop formation.

### RuvC active site arginines contribute to NTS and TS cleavage efficiencies and determining the cleavage position

To evaluate the effect of arginine substitutions on the efficiency of cleaving NTS and TS strands, we performed 6-carboxyfluorescein (FAM)-labeled cleavage assays using a 53-mer MCH and MM16 oligo-DNAs (Fig. S6*A*). MM16 was chosen since this position has the most impairment for mismatch DNA cleavage (Fig. 3, *A*–*F*; Table S3). Only one strand (either NTS or TS) of the double-stranded oligo was labeled at a time to independently monitor individual strand cleavage by the RuvC domain. We chose FnoCas12a^R918A^ and FnoCas12a^R918K/R921K^ for FAM-assays since they show the most mismatch discrimination based on plasmid cleavage assays (Fig. 3*F*; Table S4*B*). The results show that FnoCas12a^WT^ is the most active, cleaving 84 ± 5% and 86 ± 5% of NTS and TS, respectively, after 60 min (Fig. 4*A*; Table S5). Although Cas12a cleaves the NTS prior to the TS in a sequential manner (15, 24), the endpoint cleavage levels of both strands are similar in FnoCas12a^WT^, indicating that even though unwinding the TS from crRNA and bringing it to the RuvC active site is needed for TS cleavage, the extra steps do not impact cleavage efficiencies. There are no significant changes in the amount of DNA cleavage for both strands at the end of 60 min when active pocket arginines are substituted. FnoCas12a^R918A^ achieves 77 ± 2% and 64 ± 10% for NTS and TS cleavages, respectively, for MCH DNA (Fig. 4*B*; Table S5). FnoCas12a^R918K/R921K^ achieves 73 ± 19% and 72 ± 14% for NTS and TS cleavages, respectively, for MCH DNA at the endpoint of the reaction (Fig. 4*C*, Table S5). But significant difference is noticed for the efficiency of DNA cleavage for both these variants since the rate of cleavage for both NTS and TS cleavages are slower for the variants compared to FnoCas12a^WT^ (Fig. S7). This pattern is also observed for plasmid-based *cis* cleavage (Fig. 2*A*, C, F; Table S1). The most impairment is seen for TS cleavage by FnoCas12a^R918K/R921K^, with FnoCas12a^R918A^-TS cleavage closely following, indicating that the arginines play significant roles in unpairing TS from the crRNA and bringing TS as a ssDNA into the RuvC active site following the NTS cleavage (Fig. 4, *G*–*I*; Fig. S7).

**Figure 4:**
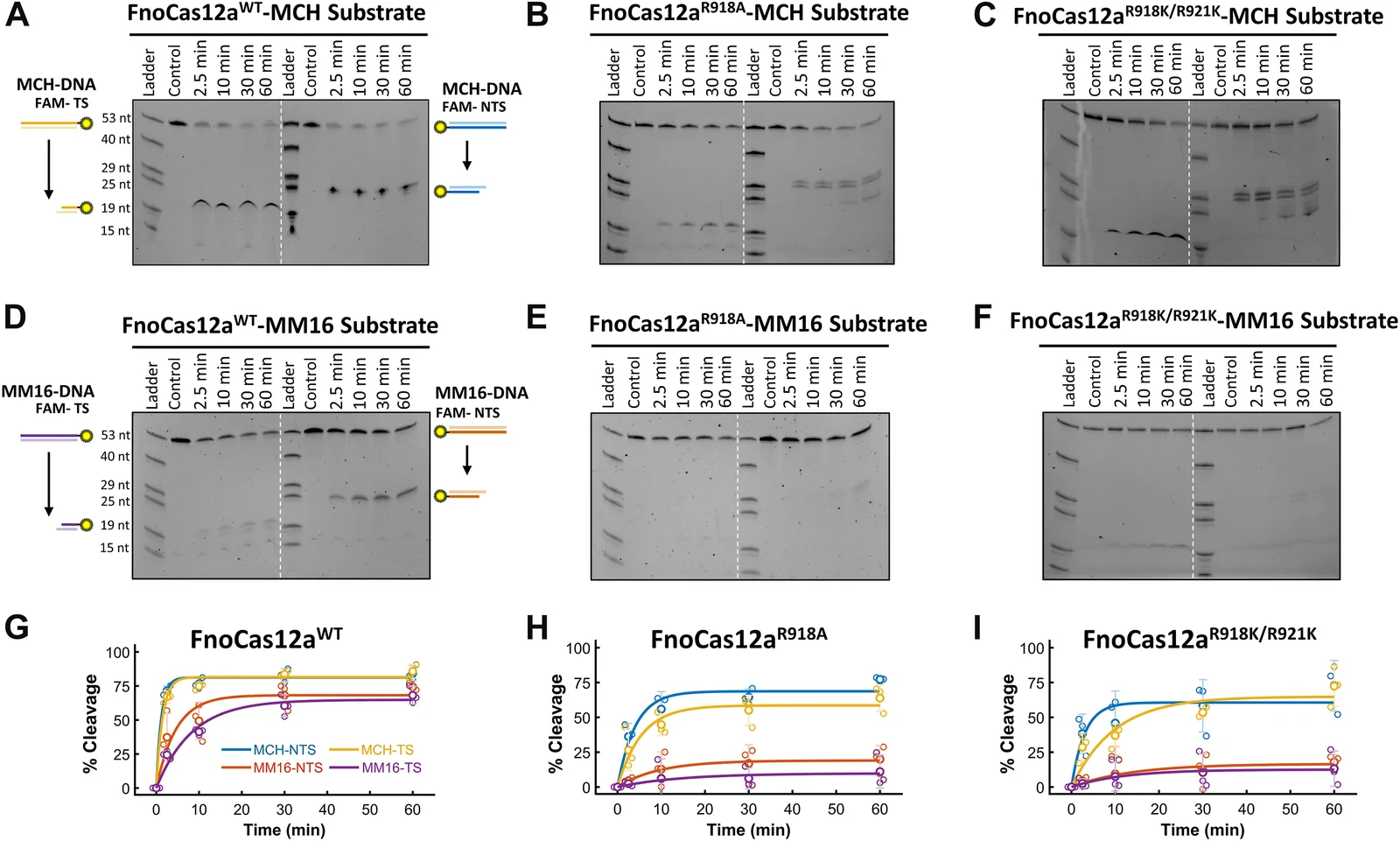
**Time-course cleavage of FAM-labeled oligonucleotide substrates by Cas12a variants.***A-C*, representative denaturing gels showing cleavage of the matched (MCH) FAM-labeled oligonucleotide substrate by FnoCas12a^WT^ (*A*), FnoCas12a^R918A^ (*B*), and FnoCas12a^R918K/R921K^ (*C*). *D-F*, representative denaturing gels showing cleavage of the MM16 mismatched substrate by FnoCas12a^WT^ (*D*), FnoCas12a^R918A^ (*E*), and FnoCas12a^R918K/R921K^ (*F*). For all panels, the *left side* of each gel shows cleavage of the TS-labeled substrate, which produces a 21-nt product corresponding to cleavage between the 21st and 22nd nt downstream of the PAM. The *right side* shows cleavage of the NTS-labeled substrate. FnoCas12a^WT^ primarily produces a 25-nt NTS product, corresponding to cleavage between the 14th and 15th nt downstream of the PAM, whereas FnoCas12a^R918A^ and FnoCas12a^R918K/R921K^ accumulate a larger 29-nt NTS product, corresponding to cleavage between the 18th and 19th nt downstream of the PAM. Control lanes contain the corresponding FAM-labeled substrate without enzyme and indicate the position of the uncleaved full-length oligonucleotide. We see trimmed product for NTS cleavage for FnoCas12a^WT^ (25-nt), while a majority of NTS cleavage product is 29-mer for the two variants. *G-I*, quantification of efficiencies of NTS and TS cleavages and DNA mismatch discrimination. Fluorescence intensity of substrate and product were quantified to determine % of product formation. Data points represent mean ± SD from three replications. The lines show data fitted using a nonlinear least-squares regression model in MATLAB and the curves show the relative efficiencies of NTS and TS cleavages and the discriminatory effect compared to MCH oligo cleavage efficiency. Only two variants, FnoCas12a^R918A^ and FnoCas12a^R918K/R921K^, and one mismatch position (MM16) were selected for this analysis since the plasmid cleavage assays indicated these variants show the highest DNA mismatch discrimination with the biggest impact in MM16. Each circle represents the mean value from three independent replications at the indicated time point, and error bars represent SD.

Additional differences are seen in the size of the product bands. Previous studies have reported different cleavage positions for Cas12a^WT^ orthologs: between 14–15th (24) or 18–19th nt downstream of the PAM for NTS cleavage and between 21–22nd or 23–24th nt downstream of the PAM for TS cleavage (7, 16, 41). In FnoCas12a^WT^, we observe cleavage between 14–15th nt for NTS (25-mer product) and 21–22nd nt for TS (21-mer product). In both FnoCas12a^R918A^ and FnoCas12a^R918K/R921K^, a longer fragment of approximately 29-mer (corresponds to cleavage between 18–19th nt downstream of the PAM) appears first and is gradually trimmed to ∼25-mer over time that matches the final product observed for FnoCas12a^WT^ (Fig. 4, *A*–*C*). To further understand the trimming pattern, we performed FAM-labeled MCH DNA cleavage assays (Fig. S6*B*) using the single substituents FnoCas12a^R918K^ and FnoCas12a^R921K^ along with other variants. The results with this single time point assay of 60 min show that both FnoCas12a^R918K^ and FnoCas12a^R921K^ produce similar sized products for both NTS and TS cleavage as FnoCas12a^WT^ (Fig. S7*B*). In addition, we performed FAM-labeled MCH DNA cleavage assays at 25 °C for 60 min using FnoCas12a^WT^ to test if we can capture a longer cleavage product at a lower temperature (Fig. S6*C*). The NTS cleavage band remains the same at lower temperature indicating that NTS trimming occurs very rapidly after the initial cleavage between 18 to 19 nt of NTS in FnoCas12a^WT^ or that with intact arginines, the protein cleaves only at the 14th position.

Overall, these results indicate that presence of R918 and R921 in the RuvC active pocket helps to facilitate the conformational changes for cleaving NTS and TS strands efficiently and to create the same length of product as FnoCas12a^WT^. On the other hand, replacing both arginines with lysine or substituting R918 with alanine negatively impacts the conformational and catalytic properties needed for DNA cleavage. These results highlight the importance of multivariate interactions possible by an arginine residue to be critical in RuvC’s catalytic activity.

We performed assays with FAM-labeled MM16 oligo DNA (Fig. S6*A*) to assess how the RuvC active site arginines contribute to cleavage efficiency of individual strands of a mismatched DNA. FnoCas12a^WT^ shows a reduced efficiency in cleaving MM16 DNA with endpoint cleavage activity of 74 ± 2% for MM16-NTS and 68 ± 7% for MM16-TS, which shows 10% and 18% reduction compared to MCH-NTS and MCH-TS cleavages respectively (Fig. 4*D*; Table S5*A*). The FnoCas12a^R918A^ and FnoCas12a^R918K/R921K^ variants exhibit a substantial loss of cleavage activity for both NTS and TS, with final cleavage of 20 ± 10% (MM16-NTS) and 11 ± 12% (MM16-TS) for FnoCas12a^R918A^, and 18 ± 5% (MM16-NTS) and 13 ± 12% (MM16-TS) for FnoCas12a^R918K/R921K^ (Fig. 4, *E* and *F*; Table S5, *B* and *C*). Relative to FnoCas12a^WT^, FnoCas12a^R918A^ variant shows 73% and 84% lower MM16-NTS and MM16-TS cleavages, respectively, while FnoCas12^R918K/R921K^ variant displays 75% and 81% reductions in MM16-NTS and MM16-TS cleavages.

Overall, these two variants have similar reductions in their efficiencies to cleave both strands of MM16 oligo DNA and demonstrate higher selectivity compared to FnoCas12a^WT^. There are slight differences in MM16 cleavage of oligo and plasmid substrates since efficient nicking is present for MM16 supercoiled plasmid cleavage assays (Fig. 3, *A*–*E*; Table S3). Since Cas12a has a sequential cleavage, in supercoiled plasmids, FnoCas12a^R918A^ and FnoCas12a^R918K/R921K^ achieve more than 90% NTS cleavage, but with very limited (∼1%) TS cleavage (Table S3). These results show that single-stranded regions in supercoiled DNA helps to negate some of the disadvantages introduced by the arginine substitutions and enable efficient NTS cleavage with severely impaired TS cleavage. In the case of oligo DNA, there is no such discrimination of NTS *versus* TS strands implying that arginine substitutions make it harder for the resulting variants to open the dsDNA oligo to enable pairing with the crRNA in the case of NTS cleavage and to unpair TS from crRNA to bring TS in a ssDNA form to the RuvC active site.

### RuvC active site arginines are essential for Cas12a′s *trans* cleavage activity

Several Cas12 family proteins exhibit *trans* cleavage activity, which forms the basis for using Cas12a as a molecular diagnostic tool (8, 42). However, although this activity is beneficial for detection assays, it is considered undesirable in genome editing applications because it can degrade ssDNA donor templates used for homology-directed repair (HDR), thereby lowering overall editing efficiency (11, 33, 34). We investigated if the arginine substitutions that we introduced to FnoCas12a′s RuvC active site pocket impact *trans* cleavage. To test this, we performed a time-course assay with a 20-nt ssDNA activator, that is fully complementary to the guide region of the crRNA, without a PAM sequence to initiate *trans* cleavage activity (8, 17). FnoCas12a^WT^ completely degrades the M13mp18 ssDNA plasmid substrate through *trans* cleavage within 1 min at 37 °C, with a calculated rate constant for substrate depletion of 2.07 ± 0.90 min^−1^ (Fig. 5, *A* and *B*; Table S6). FnoCas12a^R918K^ exhibits minimal *trans* cleavage activity, with 95 ± 3% of the substrate remaining after 60 min. Due to the minimal substrate loss and the resulting deviation from exponential decay, a rate constant could not be determined. FnoCas12a^R918A^ also shows a similar limited level of *trans* activity, retaining 97 ± 7% of the substrate at the 60-min time point (Fig. 5, *C* and *D*; Table S6). Thus, both substitutions produce comparable reduction in *trans* cleavage activity. This indicates that the increased positive charge and capacity for multiple stabilizing interactions provided by arginine at position 918 are critical, as replacement of arginine with lysine renders the enzyme largely incapable of catalyzing *trans* cleavage. This is different from *cis*-cleavage since a lysine substitution does not abolish crRNA-guided DNA cleavage.

**Figure 5:**
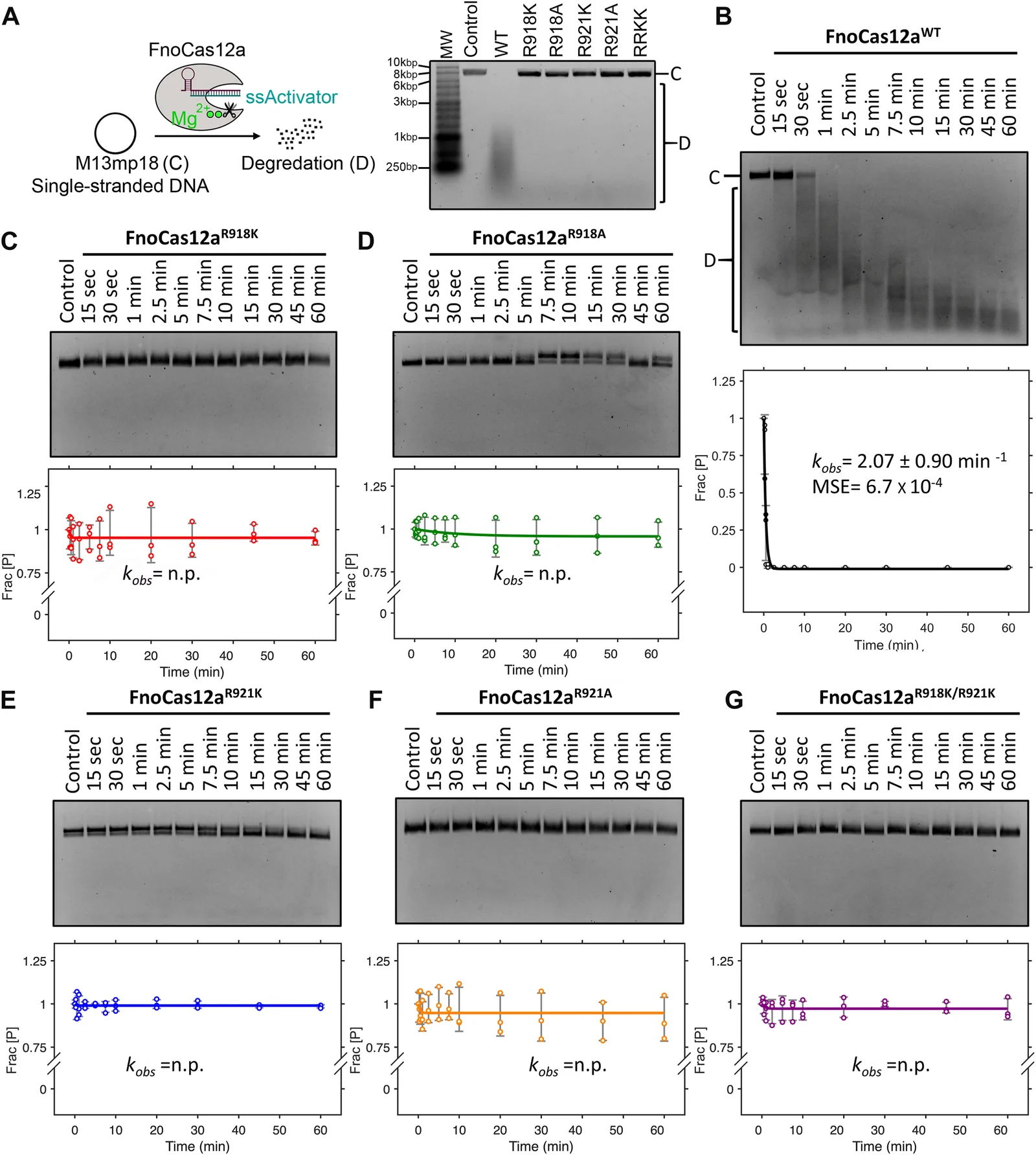
**Representative gels showing *trans* cleavage activity of FnoCas12a^WT^ and variants over time.***A*, schematic representation of the *trans* cleavage assay and representative endpoint alkaline agarose gel after 60 min. M13mp18 circular ssDNA was incubated with FnoCas12a-crRNA variants in the presence of ssDNA activator. The products were resolved on a 1% alkaline agarose gel to assess DNA size devoid of ssDNA secondary structure. A 1 kbp dsDNA ladder was used as the molecular weight (MW) marker. The corresponding native agarose gel for the endpoint *trans* cleavage assay shown in *panel A* is provided in Figure S8*A*. Note that secondary structure of ssDNA present in native gel is absent in alkaline gels. *B-G*, representative native agarose gels showing ssDNA degradation over time by FnoCas12a^WT^ (*B*), FnoCas12a^R918K^ (*C*), FnoCas12a^R918A^ (*D*), FnoCas12a^R921K^ (*E*), FnoCas12a^R921A^ (*F*), and FnoCas12a^R918K/R921K^^, RRKK^ (*G*). Control lanes contain M13mp18 ssDNA with Cas12a-crRNA without ssDNA activator. C indicates intact circular ssDNA, and D indicates degradation products from *trans* cleavage. Closely migrating bands near the intact substrate position were quantified together as remaining ssDNA substrate. Quantified substrate degradation was fitted to a single exponential decay model using MATLAB. Data points show the mean ± SD from three replicates. Solid lines represent the best-fit curves. *k*_obs_ and MSE values are shown for each variant. n.p. indicates that *k*_obs_ was not provided because MATLAB could not reliably determine the value.

At position 921, the lysine substitution in FnoCas12a^R921K^ results in 99 ± 2% substrate retention, while FnoCas12a^R921A^ leaves 91 ± 12% of the substrate intact. The double-lysine variant in FnoCas12a^R918K/R921K^ also shows minimal *trans* activity, with 97 ± 7% of substrate remaining after 60 min (Fig. 5, *E*–*G*; Table S6). Comparison of the double substitution variant with the corresponding single substitutions indicates that R918 and R921 contribute similarly to promoting *trans* cleavage, as all engineered variants exhibit comparable levels of substrate retention within experimental error. The results also show that R918 and R921 contribute differently to *cis* and *trans* cleavages, with a strict requirement for an arginine, rather than a positive charge, at these positions to support *trans* cleavage.

### Cas12a′s RuvC active site arginines promote crRNA-Independent (R-I) DNA cleavage

Previous studies have shown that Cas12a can cleave DNA in the absence of the crRNA, which is enhanced by divalent ions such as Mn^2+^ (activity called as RNA-Independent, R-I, DNA cleavage) (9, 13, 43). The R-I activity of FnoCas12a was evaluated by incubating M13mp18 single-stranded plasmid DNA with FnoCas12a and 10 mM MnCl_2_, in the absence of crRNA. A time-course assay, spanning 15 s to 1 h, was conducted to monitor the degradation over time. FnoCas12a^WT^ leaves 2 ± 2% of the substrate uncleaved following R-I cleavage within 10 min at 37 °C, with a calculated rate constant, *k*_obs_, of 2.41 ± 0.23 min^−1^ (Fig. 6, *A* and *B*; Table S7; Table S8). The FnoCas12a^R918K^ variant leaves 5 ± 2% of the substrate uncleaved after 60 min, indicating the reduction in the ability of the variant to catalyze R-I cleavage. The calculated *k*_obs_ for FnoCas12a^R918K^ is 0.08 ± 0.04 min^−1^, representing a 30-fold reduction in R-I activity compared to that in FnoCas12a^WT^. Notably, the reduction in the rate constant for R-I cleavage between FnoCas12a^R918K^ and FnoCas12a^WT^ is 4.5 times higher than the corresponding reduction for *cis* cleavage for these two proteins (6.6-fold), indicating a preference to remove non-specific DNA cleavage at a higher level than *cis* DNA cleavage. The R918K substitution could not completely eliminate R-I activity compared to the abolishment of *trans* activity by this substitution, implicating a different mechanism of binding or catalysis for these two distinct non-specific activities (Fig. 5*C*; Fig. 6*C*). For FnoCas12a^R918A^, a similar reduction in R-I activity is observed, with 9 ± 9% of the substrate remaining after 60 min. Additionally, after 1 min, 94 ± 3% of the substrate remains, indicating a significant activity reduction compared to FnoCas12a^WT^ (Fig. 6*D*; Table S7). The calculated *k*_obs_ is 0.05 ± 0.02 min^−1^, corresponding to a 48-fold reduction in R-I activity compared to FnoCas12a^WT^. Overall, alanine substitution at position R918 has a higher (1.6-fold) impairment in R-I activity compared to the lysine substitution (Table S8).

**Figure 6:**
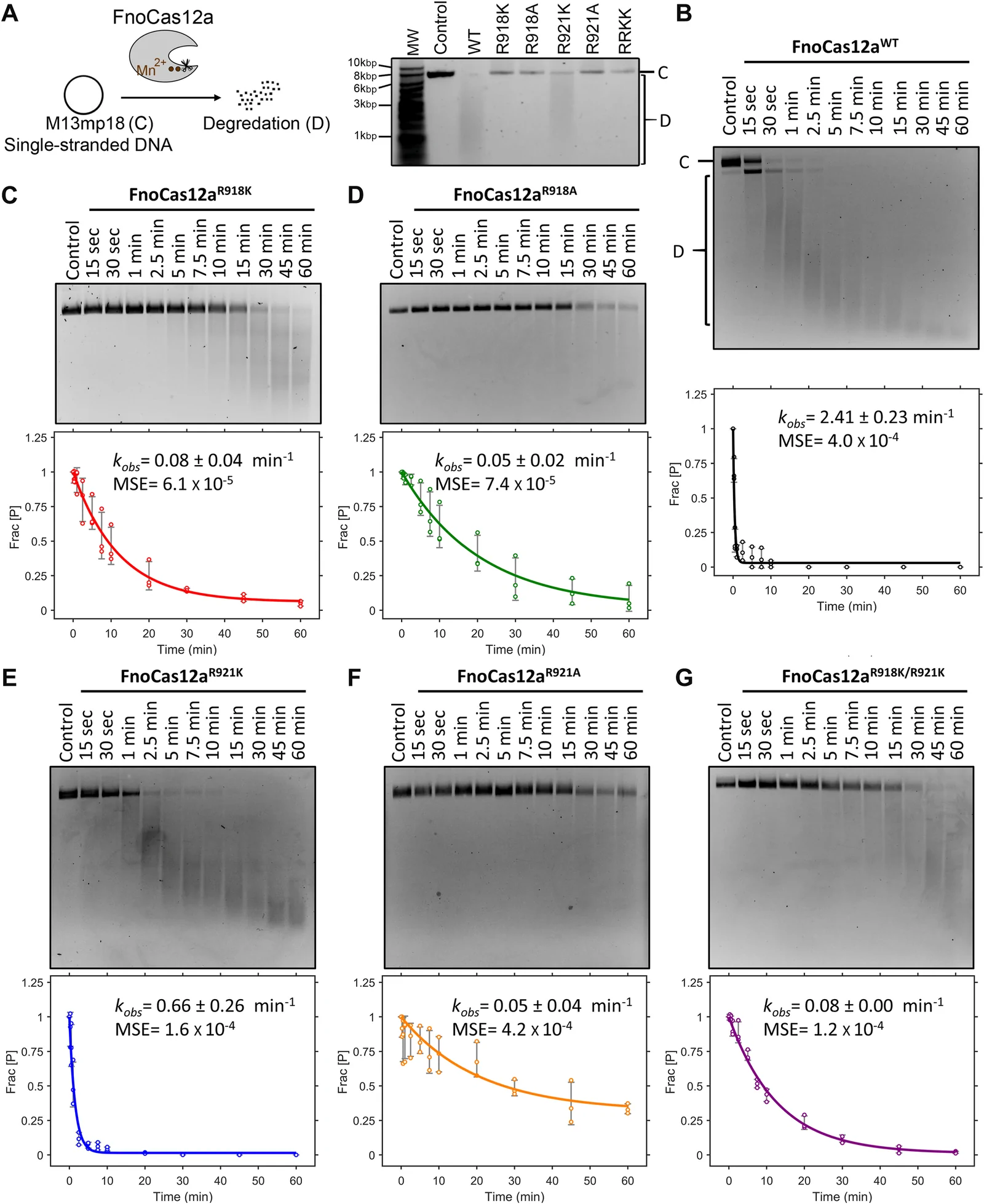
**RNA-independent (R-I) DNA cleavage kinetics for circular ssDNA substrate.***A*, schematic representation of the R-I DNA cleavage assay and representative alkaline agarose gel after 15 min of reaction. M13mp18 circular ssDNA was incubated with FnoCas12a variants in the absence of crRNA and in the presence of Mn^2+^. The products were resolved on a 1% alkaline agarose gel to assess DNA size devoid of ssDNA secondary structure. A 1 kbp dsDNA ladder was used as the molecular weight (MW) marker. The corresponding native agarose gel is provided in Figure S8*B*. Note that secondary structure of ssDNA present in native gel is absent in alkaline gels. *B-G*, representative native agarose gels showing R-I ssDNA degradation over time by FnoCas12a^WT^ (*B*), FnoCas12a^R918K^ (*C*), FnoCas12a^R918A^ (*D*), FnoCas12a^R921K^ (*E*), FnoCas12a^R921A^ (*F*), and FnoCas12a^R918K/R921K^^, RRKK^ (*G*). Control lanes contain ssDNA without enzyme. C indicates intact circular ssDNA, and D indicates degraded products. Quantified substrate degradation was fitted to a single exponential decay using MATLAB. Data points show the mean ± SD from three replications. Solid lines represent the best-fit curves. *k*_obs_ and MSE values are shown for each variant.

The R-I activity of FnoCas12a^R921K^ remains largely similar to that of FnoCas12a^WT^, with 4 ± 2% of the substrate remaining after 10 min. Comparing the initial time points, FnoCas12a^R921K^ has slower initial cleavage, with the variant catching up to FnoCas12a^WT^ within 2.5 min into the reaction, which is different from all the other variants (Fig. 6*E*; Table S7). The calculated *k*_obs_ is 0.66 ± 0.26 min^−1^, representing only a 3.6-fold reduction compared to FnoCas12a^WT^ (Table S8). These results suggest that retaining the positive charge at position 921, even with lysine is sufficient to sustain R-I activity.

In contrast, the alanine substitution in FnoCas12a^R921A^ significantly diminishes R-I activity, leaving 33% ± 3% of the substrate after 60 min which is the highest substrate retainment among the variants. The *k*_obs_ for FnoCas12a^R921A^ is 0.05 ± 0.04 min^−1^, representing a 48-fold reduction compared to FnoCas12a^WT^ (Fig. 6*F*; Table S8). These findings highlight the critical role of the positive charge at position 921 in promoting R-I activity, and its removal dramatically impairs this function. Surprisingly, FnoCas12a^R921A^, which is catalytically inactive for both *cis* DNA cleavage and *trans* activity, exhibited R-I activity. This observation suggests that the mechanisms governing *cis* and *trans* activities differ fundamentally from that underlying R-I activity.

Comparing the contributions of the two arginines, the presence of arginine in position 918 is more critical than 921 as R921K leaves 4% ± 2% of the substrate remaining after 10 min, whereas R918K leaves 47% ± 14% remaining. Alanine substitutions have a different effect with R921A having a slightly higher impact based on slower degradation pace and accumulation of R-I substrate in FnoCas12a^R921A^ compared to that in FnoCas12a^R918A^ (Fig. 6, *D* and *F*). This effect is conserved for *cis* cleavage as well with FnoCas12a^R921K^ having a similar rate constant to FnoCas12a^WT^ for *cis* cleavage, and FnoCas12a^R921A^ having complete absence of *cis* cleavage compared to very slow, but efficient DNA cleavage, in FnoCas12a^R918A^.

R-I activity of double lysine substitutions in FnoCas12a^R918K/R921K^ shows just 2% ± 0% remaining substrate after 60 min, with the degradation pattern closely following that of FnoCas12a^R918K^ (Fig. 6*G*; Table S7). The *k*_obs_ is 0.08 ± 0.00 min^−1^, corresponding to a 30-fold reduction in R-I cleavage efficiency compared to that of FnoCas12a^WT^ (Table S8). Interestingly, a comparison of the rate constants for single and double lysine substitutions show that the major impact for R-I activity comes from R918 since the value is similar to that from a single lysine substitution at R918. Overall, these results show that while arginines in the RuvC active site are important for DNA cleavage, there is positional difference in their relative contributions. A positive charge from a lysine at position R921 is not detrimental for R-I activity compared to an alanine substitution, while a mere positive charge from a lysine at position 918 is not sufficient with an almost similar detrimental effect on R-I activity with both lysine and alanine substitutions.

### Differential structural contributions of R918 and R921 to FnoCas12a catalytic site organization

Through performing Molecular Dynamics (MD) simulations and carrying out subsequent solvent-accessible surface area (SASA) analysis and principal component analysis (PCA), we assessed whether residues R918 and R921 and their substitutions make distinct structural contributions to the FnoCas12a catalytic site. The pre-catalytic model of FnoCas12a was built using the cryo-EM structure available under PDB ID: 6GTG (24), with DNA and Mg^2+^ ions coordinates taken from an AsCas12a structure, PDB ID: 8SFO (25) to obtain the substrate-bound activated-state configuration. After 1 μs of MD production simulation (see Section S1 in the Supporting Information for details) and confirming model stability (Fig. S9), we focused our analysis on the local environment surrounding residues R918 and R921. Since these residues are positioned near both the RuvC catalytic pocket and the NTS-DNA, our computational analysis was centered on local structural rearrangements around the catalytic site rather than global protein motions.

We analyzed two distances to monitor the organization of the catalytic site: (#1) the distance between the Cβ atom of residue 918 (arginine, lysine or alanine) and the Cδ atom of Glu1028 in the downstream helix, and (#2) the distance between the Cβ atom of residue 921 and the phosphate group of the 19th nucleotide in the NTS (counted from the PAM and located adjacent to the reported cleavage site at the 18th nucleotide) (Fig. 7*A*). The first distance was used to probe the relationship between residue 918 and the region surrounding Glu1028, which is associated with the RuvC-lid and downstream helix element involved in catalytic site organization (Fig. 1, *B* and *C*; Fig. S2). The second distance was used to evaluate NTS positioning near residue 921, where direct interactions with the DNA backbone are observed close to the scissile phosphate (Fig. 1*E*; Fig. S3). The results show that FnoCas12a^WT^ and FnoCas12a^R921K^ access similar configurations in the two-dimensional distance space analyzed, and thus preserve comparable arrangements between NTS, residue 921, and residue 918 (Fig. 7*B*). This is consistent with preservation of a productive active site configuration when a positive charge is retained at position 921.

**Figure 7:**
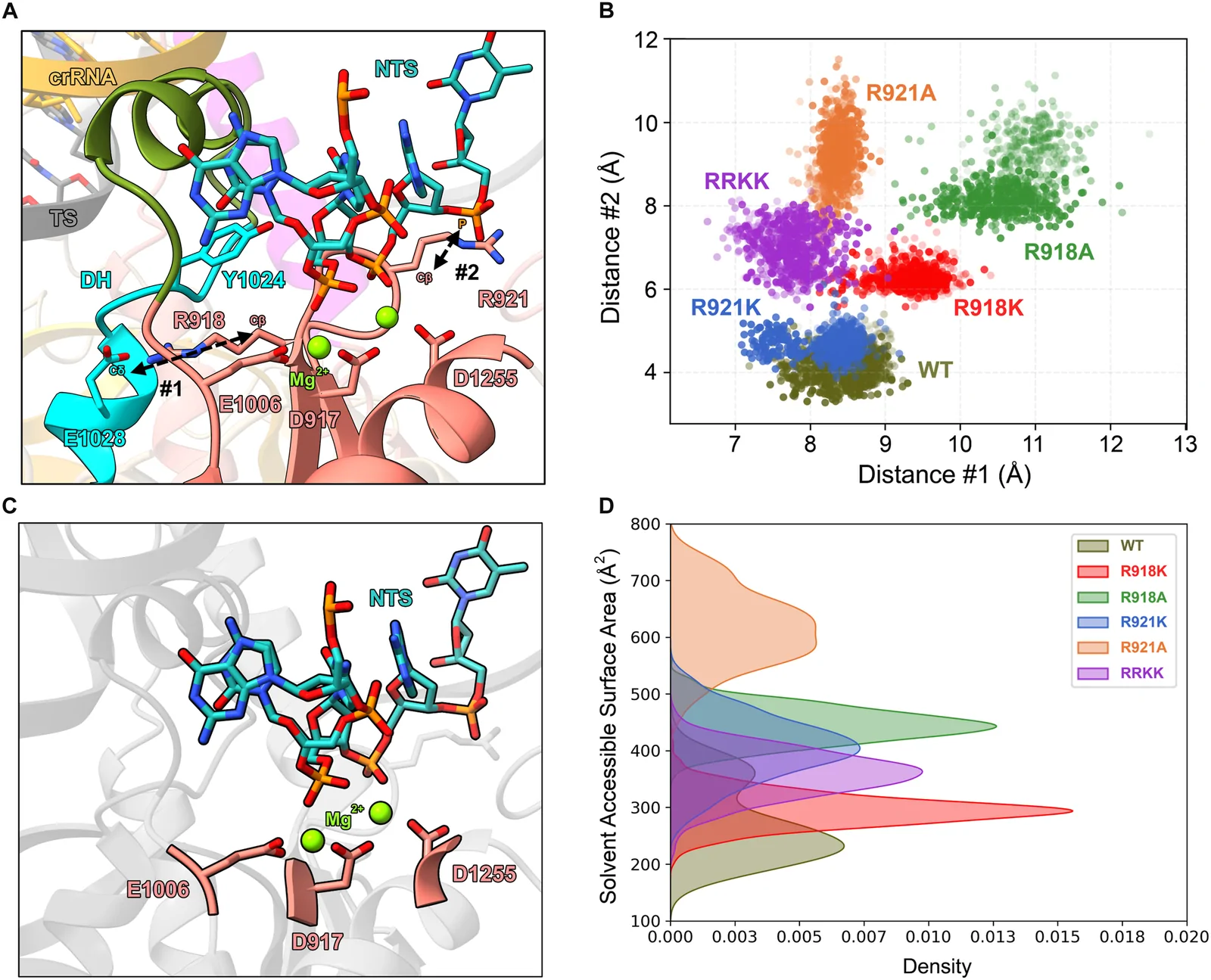
**Structural and molecular dynamics analyses of the RuvC active-site arginine variants.***A*, structural model of the FnoCas12a RuvC active pocket showing the positions of R918 and R921 relative to the catalytic residues, Mg^2+^ ions, and the NTS-DNA substrate. Distance #1 corresponds to the distance between the Cβ atom of R918 and the Cδ atom of E1028, while Distance #2 corresponds to the distance between the Cβ atom of R921 and the phosphate group of the 19th nt of NTS, adjacent to scissile phosphate. [DH: downstream helix (*cyan ribbon*), NTS: non-target strand (turquoise sticks), RuvC lid: *green*, RuvC: *salmon*]. *B*, Scatter plot showing the distribution of Distance #1 and Distance #2 sampled during molecular dynamics simulations for FnoCas12a^WT^ and the indicated variants. *C*, structural representation of the residues, Mg^2+^ ions, and nucleotides included in the solvent-accessible surface area (SASA) analysis of the RuvC active-site pocket is shown in colored region. The *gray* region is excluded from SASA analysis. [NTS: non-target strand (turquoise sticks), Catalytic residues in RuvC: *salmon*]. *D*, the SASA values calculated for the RuvC active-site pocket during molecular dynamics simulations of FnoCas12a^WT^ and the indicated variants. (RRKK, FnoCas12a^R918K/R921K^). As the SASA values increase, the activity decreases.

In contrast, FnoCas12a^R921A^ is mainly shifted along the coordinate related to NTS positioning near residue 921 (Fig. 7*B*), supporting the idea that R921 contributes directly to positioning the NTS phosphate backbone near the catalytic site. The R918 variants affect both distance coordinates, with FnoCas12a^R918K^ showing intermediate behavior and FnoCas12a^R918A^ strongly deviating from FnoCas12a^WT^ (Fig. 7*B*). Interestingly, substitutions at R918 change both structural coordinates, whereas those at R921 mainly affect the coordinate related to NTS positioning, where a direct interaction is seen with the DNA. FnoCas12a^R918K/R921K^ also occupies a distinct conformational region and follows the same trend observed for the R918-containing variants (Fig. 7*B*). These observations suggest that residue R918 functions in an earlier step of the catalytic pocket organization, potentially by supporting the folding or positioning of the RuvC-lid and downstream helix needed to open and organize the active site for proper NTS positioning. In this model, R921 acts more locally by helping stabilize the NTS backbone near the scissile phosphate. As a result, changes at R918 can influence both structural distances, while changes at R921 remain more localized and do not strongly affect the Glu1028-R918 coordinate. These observations support different structural roles for the two residues during catalytic-site organization.

This interpretation is supported by the SASA analysis of the active site region, which included the catalytic residues, nearby nucleotides, and both catalytic Mg^2+^ ions (Fig. 7*C*). SASA was used to evaluate how substitutions at R918 and R921 affect solvent exposure within the catalytic pocket, an important property because productive catalysis requires an active site that is accessible enough to accommodate the nucleic acid substrate and catalytic water molecules but not so exposed that the substrate and catalytic residues become poorly organized. The SASA analysis results generally follow the trend observed for distance #2, which reflects NTS positioning relative to residue 921. Lower SASA values indicate reduced solvent exposure and are consistent with closer NTS-protein interactions. FnoCas12a^WT^ shows the lowest solvent accessibility (Fig. 7*D*), suggesting stronger stabilization of the NTS near the catalytic region, which may contribute to its higher cleavage activity (Fig. 2*A*; Table S2). In contrast, alanine substitutions increase distance #2 (Fig. 7*B*) and are associated with increased solvent exposure (Fig. 7*D*), consistent with reduced NTS-protein interactions and impaired catalytic activity observed for FnoCas12a^R921A^ and the reduced cleavage rate observed for FnoCas12a^R918A^ (Table S2). The double lysine substitutions (FnoCas12a^R918K/R921K^) produce smaller changes in distance #2 (Fig. 7*B*) and SASA (Fig. 7*D*), potentially because retention of a positively charged side chain partially preserves interactions that maintain local structural organization. Comparing this result with the rate constants of *cis* cleavage (Fig. 2*G*) indicates that productive catalysis may require a balanced level of active-site accessibility, and that increased solvent exposure can shift the system away from the most efficient catalytically competent state.

PCA was then used to evaluate whether the local rearrangements observed in the distance and SASA analyses were associated with distinct collective motions of the catalytic region. PCA reduces the dimensionality of the MD trajectories by identifying dominant correlated motions, with PC1 and PC2 representing collective movements with the largest amplitudes that were sampled during the simulations (44). Here, PCA was focused on atoms surrounding the catalytic site (shown in Fig. S10*A*), including protein backbone atoms, nucleic acid atoms, and catalytic Mg^2+^ ions, so that the analysis captured local active site dynamics rather than global motions of the full Cas12a complex.

PC1 further orders the systems along a WT → R921K → R918K/R918K-R921K → R918A/R921A progression (see the PC1 histogram at the top of Fig. S10*B*). This progression is in general consistent with distance #2 (Fig. 7*B*) and SASA analyses (Fig. 7*D*) and suggests that the substitutions progressively shift the catalytic pocket away from the wild-type-like conformational ensemble. The dominant motion along PC1 involves rearrangement of the RuvC-lid and downstream helix together with changes in the openness of the catalytic pocket (Fig. S10*C*, Movie S2), whereas PC2 captures additional local fluctuations around the active site region (Fig. S10*D*; Movie S3).

In addition, RMSD and RMSF analyses of the protein, crRNA, NTS, and TS do not show major global structural changes between the systems (Fig. S9; Fig. S11; Fig. S12). RMSD was used to monitor the overall structural stability of each simulated complex, whereas RMSF was used to identify residue- or nucleotide-level flexibility along the trajectories. The absence of major global differences indicates that the observed changes are not caused by large-scale destabilization of the Cas12a complex but instead arise mainly from local rearrangements within the catalytic pocket. Together, these results provide additional structural insights into the importance of arginines in the active pocket. R918 contributes to organizing the active site pocket by supporting the RuvC-lid and downstream helix arrangement, and changes in this residue mainly weaken the conformational framework that supports efficient and specific DNA cleavage. R921 is important for catalytic competence driven by positioning the NTS in the catalytic pocket and maintaining a balanced solvent accessibility.

## Discussion

### Distinct roles for R918 and R921 in regulating *cis* DNA cleavage of FnoCas12a

The *cis* cleavage data reveals distinct roles for R918 and R921 in controlling the catalytic activity of FnoCas12a. Structural analysis suggests that R918 participates in conformational transitions of the RuvC-lid (PDB IDs: 6I1K and 6GTG) (11, 24), whereas R921 contributes to positioning of the NTS-DNA scissile phosphate near the catalytic center (Fig. 1; Fig. S2). Our biochemical data support these structural roles since R918 substitutions primarily modulate cleavage efficiency and kinetics, while those for R921, especially R921A, make the variant inactive for *cis* cleavage (Fig. 2*E*), implying a role in directly supporting catalysis. Since substitution of R918 with lysine or alanine only slows DNA linearization without eliminating the activity (Fig. 2, *B* and *C*), it indicates that R918 is not directly participating in phosphodiester bond cleavage. The reduced linearization rates, prolonged accumulation of nicked intermediates (Fig. 2, *B* and *C*), and the structural interactions seen for R918 (Fig. 1, *A* and *B*) suggest that R918 fine-tunes catalysis by stabilizing productive RuvC-lid conformations that promote efficient substrate access and processing. In contrast, R921 plays a critical role in *cis* cleavage. While the R921K substitution retains partial activity (Fig. 2*D*; Table S1) with rate constants closer to FnoCas12a^WT^ (Table S2), substitution with alanine completely abolishes *cis* cleavage (Fig. 2*E*; Table S1). This strict requirement for a positively charged residue at position 921 is consistent with its proposed role in stabilizing the NTS strand in the vicinity of the scissile phosphate and supporting the catalytic chemistry at the RuvC active site. It is also interesting to note that FnoCas12a^R921K^ traps part of the complex in an inactive conformation since, after the fast achievement of 66% linearization within 1 min into the cleavage reaction, the remaining DNA stays as a mixture of supercoiled substrate and nicked intermediate (Fig. 2*D*; Table S1). Currently available structures do not provide mechanistic insights into this behavior.

The reduction in cleavage rate observed for the double lysine variant (FnoCas12a^R918K/921K^, Table S2) further highlights the coordinated contributions of R918 and R921. Despite preserving positive charge at both positions, the combined substitutions reduce *cis* cleavage more than the single lysine substitutions. This indicates that precise side chain interactions, rather than charge alone, are required for optimal catalysis. Together, these findings demonstrate that R918 acts as a regulatory residue that fine-tunes DNA cleavage efficiency, whereas a positive charge at position 921 is indispensable for *cis* cleavage. This is further supported by amino acid sequence conservation of R918 and R921. Of the 91 different Cas12a sequences that we analyzed, all but one is conserved as Arg at position 918. For 921, there is a good amount (29%) of distribution of lysine in place of arginine, and two instances (2%) where arginine is replaced with asparagine (Fig. 1*D*; Fig. S1). The amino acid conservation pattern is also supported by our MD data that show that R918A substitution impacts both RuvC-lid ordering and NTS positioning, while R921A impacts only NTS positioning (Fig. 7*B*), requiring stronger conservation of Arg at position 918. Thus, our results provide insights into protein engineering strategies for developing Cas12a orthologs with higher DNA cleavage fidelity.

Previous structural analysis proposed that positively charged residues in the proximity of RuvC active pocket, R918 and K1013 in FnoCas12a, contribute to a molecular lock of the RuvC-Nuc pocket through polar interactions with catalytic residues E1006 and D917, respectively, thereby occluding the catalytic site prior to DNA cleavage (24). While this model explains regulated access to the active site, we propose that the role of R918 extends beyond a locking function. Structural comparisons of R909 of AsCas12a in a series of structures with different R-loop formation (Fig. S2) show that R918 of FnoCas12a can potentially engage in multiple interactions with residues within the RuvC-lid and the downstream helix, with interacting residue pairs between the two elements changing before and after the RuvC-lid folds into an α-helical conformation as the complex transitions through R-loop formation and activation for DNA cleavage (Movie S1). These interactions indicate that R918 may facilitate and stabilize the conformational organization of the RuvC-lid and its coupling to downstream structural elements, thereby promoting proper active-site architecture rather than merely restraining access of DNA to the active pocket. Since R918 contributes to conformational changes, rather than catalytic state, both lysine and alanine substituents are active, with more impairment in the alanine substituent (Fig. 2, *B* and *C*; Table S1).

In addition, previous studies have shown that basic and polar residues, particularly arginines play critical roles in regulating on-target cleavage by stabilizing DNA substrates and enabling productive catalytic conformations. Electrostatic interactions between such residues and the NTS are important for positioning the NTS in the binding channel within the RuvC active site. In AsCas12a, residues R912, K949, K1072, R1127, R1172, R1226, and N1295 were reported to form a network that stabilizes and positions the displaced NTS across the RuvC catalytic pocket (25). Superposing AsCas12a′s DNA position (PDB ID: 8SFO (25)) into FnoCas12a’s ternary structure (PDB ID: 6GTG (24)) reveals that the positively charged and polar residues lining the NTS-binding channel are mainly conserved between the two nucleases, with R921, K962, K1085, K1136, R1153, R1218, and N1288 being the corresponding residues in FnoCas12a (Fig. S3). Among these, residues R921 and R1218 are closest to the RuvC active pocket (Fig. S3) and in a previous study it was shown that R1218 contributes to positioning the DNA substrate for hydrolysis in the catalytic pocket and that its replacement with alanine abolishes *cis* cleavage (12, 36). Consistent with the importance of electrostatic interactions in NTS positioning, our mutational analysis further shows that maintaining a positively charged residue at position 921 is important for *cis* cleavage activity, since the R921K substitution retains partial activity whereas the R921A substitution abolishes cleavage (Fig. 2, *D* and *E*). The loss of DNA cleavage caused by the R921A substitution in FnoCas12a, together with the retainment of activity in the R921K variant, suggests that the positive charge at this position is indispensable for proper positioning and/or catalysis of the NTS.

### Active-site electrostatics modulate mismatch tolerance by impacting the sequential strand cleavage process

Mismatch discrimination by Cas12a is governed not only by its ability to form a stable R-loop when mismatches are present in the substrate, but also on the enzyme’s capability to complete the sequential NTS and TS cleavages. The strongest improvements in mismatch discrimination are observed for the double lysine variant, FnoCas12a^R918K/R921K^ and FnoCas12a^R918A^, where DNA linearization is reduced when mismatches are present in the substrate. This effect is most pronounced in positions MM13-MM17, a region where R-loop destabilization is known to impede cleavage progression (17). In these regions, discrimination levels exceed those predicted from the additive effects of single lysine substitutions (Fig. 3*F*; Table S4). R918A substitution closely follows FnoCas12a^R918K/R921K^, showing higher discrimination across all these positions (Fig. 3*F*). These results indicate that modulating the electrostatic interactions of the RuvC active site with RNA/DNA can reshape the energetic landscape of cleavage, amplifying sensitivity to mismatch cleavage while retaining acceptable on-target performance. Importantly, the differential effects of lysine *versus* alanine substitutions highlight the importance of both charge magnitude and side chain geometry in tuning substrate’s mismatch tolerance.

Another interesting feature we see is the enhanced accumulation of nicked intermediates when provided with a supercoiled DNA substrate possessing mismatches, especially in positions MM13-MM17 (Fig. 3, *A*–*E*; Table S3). We see efficient first strand (NTS) cleavage and the enzyme stalling for TS cleavage, indicating that the arginines tested in this study critically contribute to unpairing TS from the crRNA and bringing it in a ssDNA form into the RuvC active site. We do not see this differential effect while using a dsDNA oilgo where Cas12a variants have to unpair the DNA strands effectively for both NTS and TS DNA cleavages (Fig. 4; Table S5). In this case, both NTS and TS cleavages are drastically reduced, without noticeable preference for one of the strands. Thus, our data show that the multivariate interactions that an arginine can provide in the RuvC active site are important in RNA-DNA pairing/unpairing, positioning the strand ideally in the active site, as well as holding the scissile phosphate intact to facilitate DNA cleavage.

### RuvC active-site arginines influence NTS positioning and scissile phosphate selection

Analysis of strand-specific cleavage using fluorescently labeled oligonucleotides shows that substitutions of R918 and R921 significantly slow down NTS and TS cleavages and impact NTS cleavage pattern, while the overall cleavage pattern for the TS does not change (Fig. 4, *A*–*C*; Table S5). The appearance of longer NTS cleavage products can potentially be due to accumulation of partially cleaved intermediates in variants compared to FnoCas12a^WT^. However, another possible explanation is that substitution of the RuvC active-site arginines changes how the NTS is positioned inside the catalytic groove. Without these arginine residues, the DNA backbone may not be held tightly, and the strand may enter the active site in different ways, leading to cleavage at different positions in different molecules in variants. This is supported by observations that only the variants produce NTS fragments with different sizes and that an activity assay with FnoCas12a^WT^ at room temperature produces the same final NTS product size even with a reduction in the cleavage rate (Fig. S7*B*). Overall, these results indicate that RuvC active-site arginines play an important role in positioning the NTS in the active pocket and thereby control where cleavage occurs in the DNA.

### Differential sensitivity toward *cis* and trans cleavages from perturbing arginine in the RuvC active pocket

The differential effects of arginine substitutions on *cis* and *trans* cleavages underscore mechanistic differences between these activities. *Cis* cleavage, which occurs as a largely single-turnover process on a stably bound R-loop substrate (8, 35, 45), is relatively tolerant to perturbations in R918 and R921, except in FnoCas12a^R921A^, which abolishes DNA cleavage. Variants, even with a markedly reduced cleavage rate constant such as FnoCas12a^R918A^ and FnoCas12a^R918K/R921K^, ultimately achieve substantial DNA linearization over extended time periods (Fig. 2). The accumulation of nicked intermediates and delayed TS cleavage point to impaired coordination between the two strand cleavage steps, yet the enzyme remains capable of completing *cis* cleavage with longer incubation times (Fig. 2; Table S1).

In contrast, *trans* cleavage is strongly impaired by all substitutions at either R918 or R921, including with lysine (Fig. 5; Table S6). This sensitivity is consistent with the requirement for repeated cycles of substrate engagement and release during *trans* activity (25, 46), which places greater demands on precise RuvC active-site organization, its prolonged and stable open state, and DNA positioning (25). Our observation that R918 substitution suppresses *trans* cleavage is consistent with previous work that showed R918G substitution in FnoCas12a abolishes *trans* degradation of ssDNA (24). Moreover, prior structural and functional analyses have demonstrated that interactions between ssDNA and residues lining the RuvC active pocket are more critical for *trans* cleavage than for *cis* cleavage. In particular, substitution of a phenylalanine residue with alanine (F999A) within the RuvC lid of AsCas12a eliminated *trans* activity while leaving *cis* cleavage largely intact (25). Together, these findings indicate that *trans* cleavage is especially sensitive to disruptions in RuvC-lid organization and substrate stabilization, whereas *cis* cleavage can proceed with reduced efficiency under the less optimal catalytic configurations introduced by the arginine substitutions.

### RNA-independent cleavage reveals a third mechanistic regime distinct from *cis* and trans activities

R-I cleavage provides a unique window into the intrinsic catalytic capacity of the RuvC domain in the absence of crRNA. Our results demonstrate that R-I activity is mechanistically distinct from both *cis* and *trans* cleavages. While substitutions at R918 strongly reduce R-I cleavage efficiency (30- and 48-fold reduction for FnoCas12a^R918K^ and FnoCas12a^R918A^, respectively, compared to FnoCas12a^WT^) (Table S8), it is not abolished, indicating that some level of catalytic activity can be retained even when conformational regulation is compromised. In contrast, R921 displayed a different sensitivity profile: retention of positive charge *via* lysine substitution preserves R-I activity (with only 3.6-fold reduction, Fig. 6*E*; Table S8), whereas alanine substitution substantially impairs it (48-fold reduction; Fig. 6*F*; Table S8). Since FnoCas12a^R921A^ has no detectable *cis* cleavage as well, the significant reduction of R-I activity should also relate to a catalytic impairment rather than a feature that can be used to engineer R-I deficient variants. Notably, FnoCas12a^R921A^, despite being inactive for both *cis* and *trans* cleavages, retained measurable R-I activity. This result indicates that the structural and chemical requirements for R-I cleavage are fundamentally different from those governing crRNA-dependent activities. The presence of Mn^2+^ may relax geometric constraints or enable alternative catalytic pathways that bypass the conformational cascade and strict substrate positioning enforced during R-loop-dependent cleavage. In fact, our previous work on an R-I-deficient SpyCas9 variant (SpyCas9^H982A^) showed similarities and differences in the placement of DNA in the RuvC active site and how H982 activates DNA cleavage through Mn^2+^-dependent stabilization of the catalytic center. In SpyCas9 where H982 facilitates removal of a Mn^2+^-coordinated water molecule to promote productive Mn^2+^ coordination with catalytic residues and positioning DNA for cleavage (47). These findings emphasize that Cas12a′s multiple nuclease activities are not simply variations of a single mechanism but rather represent distinct operational modes that differentially use the same catalytic core.

### Molecular dynamics simulations reveal local active-site remodeling by R918 and R921 substitutions

MD simulations provide a structural framework for understanding the biochemical differences between R918 and R921 variants. The pre-catalytic FnoCas12a model was generated from the PDB ID: 6GTG structure (24), with substrate DNA coordinates incorporated from PDB ID: 8SFO (25) and modified to match the 6GTG sequence. The simulations showed no major global destabilization among the variants (Fig. S9; Fig. S11; Fig. S12), indicating that the experimentally observed changes in nuclease activity likely arise from local active-site rearrangements rather than large-scale unfolding or disruption of the Cas12a complex.

Two distance coordinates were used to monitor active-site organization: the distance between residue 918 and E1028 of the downstream helix, which seems to play a role in RuvC-lid’s conformational changes, and the distance between residue 921 and the phosphate group of the 19th nucleotide in the NTS. FnoCas12a^WT^ and FnoCas12aR^921K^ occupy closely related conformational states, consistent with preservation of a productive active-site architecture when a positive charge is retained at position 921 (Fig. 7*B*). In contrast, FnoCas12a^R921A^ shifted mainly along the NTS-positioning coordinate, supporting the idea that R921 contributes directly to stabilization of the NTS phosphate backbone near the catalytic site. Substitutions at R918 affect both coordinates, with FnoCas12a^R918K^ and FnoCas12a^R918K/R921K^ showing intermediate behavior and FnoCas12a^R918A^ producing a stronger displacement from the wild-type conformational state (Fig. 7*B*).

The SASA analysis further supports this interpretation by showing that substitutions at R918 and R921 alter active-site solvent exposure (Fig. 7*D*). Overall, the SASA distribution follows the same trend observed for the NTS-positioning distance. FnoCas12a^WT^ shows the lowest solvent exposure, consistent with tighter NTS-protein contact and a more compact catalytic-site arrangement. Alanine substitutions, especially R921A and R918A, increase solvent accessibility, consistent with weaker NTS stabilization and impaired catalytic activity. In contrast, lysine substitutions produce smaller changes in solvent exposure, likely because retention of a positively charged side chain partially preserves local electrostatic interactions. These results suggest that efficient catalysis requires proper NTS positioning together with a balanced level of active-site accessibility; increased solvent exposure may reflect loss of productive substrate organization within the catalytic pocket.

PCA of the catalytic region further separates the wild-type and variant ensembles along dominant collective motions (Fig. S10). FnoCas12a^R921K^ remains closest to FnoCas12a^WT^, whereas FnoCas12a^R918K^ and FnoCas12a^R918K/R921K^ occupy intermediate regions, and FnoCas12a^R918A^ and FnoCas12a^R921A^ show the strongest deviations. The dominant motion involves rearrangement of RuvC-lid and the downstream helix together with changes in catalytic pocket’s openness (Fig. S10, *C* and *D*). Together, the MD analyses provide a mechanistic explanation for the different effects of R918 and R921 substitutions. R918 appears to support broader catalytic pocket organization, including the RuvC-lid, the downstream helix, and NTS positioning, whereas R921 acts more locally by stabilizing the NTS near the scissile phosphate. This distinction explains why R918 substitutions perturb multiple structural features, while R921 substitutions primarily affect substrate positioning within the catalytic site.

### Understanding active-site amino acid interactions will facilitate rational engineering of high-fidelity Cas12a variants

Our findings establish that arginines within the RuvC active site pocket are tunable regulators of Cas12a activity, governing conformational accessibility, substrate positioning, trimming efficiency, and mismatch tolerance. R918 position of FnoCas12a emerges as a particularly attractive engineering target, as its modification selectively suppresses *trans* and lowers R-I DNA cleavages while preserving substantial *cis* activity and markedly enhancing mismatch discrimination. In contrast, R921 or a positive charge to position the phosphate flanking the scissile phosphate is indispensable for catalysis and therefore less amenable to substitution without the complete loss of *cis* cleavage. Our MD simulation results provide further layer of distinct regulation by the two arginines, with R918 having an allosteric effect on NTS positioning through the interaction of R921 with NTS DNA (Fig. 7*B*). Even though amino acid conservation analysis shows stronger conservation for R918 compared to R921 (Fig. 1*D*; Fig. S1), our biochemical data indicates greater flexibility in different amino acid substitutions at R918 compared to R921. The MD simulations show that the conformational mechanism of RuvC domain is affected by local perturbations caused by substitutions at R918 and R921 positions. R918 substitutions affect the active site by perturbing RuvC-lid and the downstream helix organization and indirectly affecting NTS position (Fig. 7*B*), while R921 substitutions primarily affect the local placement of the NTS near the catalytic center (Fig. 7*B*). These results support the view that R918 acts as a conformational regulator, whereas R921 acts in the substrate positioning needed for the catalytic stage. These different roles could explain how alterations of R918 can tune cleavage outcomes, while removal of the positive functional group at 921 results in more severe perturbations.

Thus, our results provide a mechanistic framework that accommodates different aspects of active-pocket arginines for designing Cas12a variants optimized for genome editing applications, where minimizing off-target, *trans*, and R-I cleavages are essential, while maintaining acceptable on-target cleavage. More broadly, it highlights how fine-tuning electrostatic and conformational features within RuvC nuclease’s active site can selectively bias enzymatic outcomes across multiple functional modes.

## Experimental procedures

### Protein mutagenesis, overexpression, and purification

The wild-type *Fnocas12a* gene is used in the pET28-His8-3C-His7-MBP-TEV-FnoCas12a construct from our previous study (9). Site-directed mutagenesis (SDM) was carried out using this construct as a template to introduce single and double substitutions: R918K, R918A, R921K, R921A, and R918K/R921K (Table S9). The resulting PCR reaction, after DpnI digestion and heat inactivation, was transformed into *Escherichia coli* DH5α cells, and plasmids were subsequently isolated and sequenced to confirm the presence of the substitutions and the accuracy of the rest of the coding sequence. The verified plasmids were transformed into *E. coli* Rosetta 2 (DE3) cells for protein expression. The FnoCas12a variants were expressed and purified through a series of chromatography steps, including Ni-NTA affinity, cation exchange SP-HP, and Superdex 300 size-exclusion columns (GE Healthcare) using a previously published protocol (40).

### crRNA transcription

The template (noncoding) strand for crRNA transcription immediately downstream of the T7 promoter, and its complementary coding strand, containing only the T7 promoter, were ordered as oligo DNA from Sigma-Aldrich (Table S10). These strands were annealed at a 1:1.5 M ratio in 1X annealing buffer (10 mM Tris-HCl, pH 8, 50 mM NaCl) by heating at 95 °C for 2 min and then slowly cooling to room temperature. An *in vitro* transcription reaction (200 μL) was performed using approximately 800 ng of the annealed dsDNA in transcription buffer [1X: 40 mM Tris/HCl, pH 8.0, 1 mM spermidine, 50 μg bovine serum albumin, 20 mM MgCl_2_, 5 mM dithiothreitol (DTT)], nucleotide triphosphates (9 mM GTP, 8 mM ATP, 8 mM CTP, and 8 mM UTP), 50 μg RNasin (Promega, Madison, WI, USA), 1 μg inorganic pyrophosphatase, and 40 μg T7 RNA polymerase. The reaction was incubated for 4 h at 37 °C. The resulting transcription mixture was treated with DNase I (NEB) with final concentration of 0.01 mg/mL in 1X DNase I buffer (10 mM Tris, pH 7.5, 2.5 mM MgCl_2_, and 0.5 mM CaCl_2_) for 30 min at 37 °C to remove the template DNA. The RNA products were ethanol-precipitated and gel purified from a 12% urea (8 M) polyacrylamide gel and stored at −20 °C and annealed later according to our previously established protocol (9, 17, 40).

### Supercoiled plasmid cleavage assays

The FnoCas12a proteins were diluted to the desired concentration in a buffer containing 20 mM HEPES at pH 7.5, 150 mM KCl, 2 mM EDTA, and 1 mM TCEP. The reaction was conducted in a total volume of 10 μL in 1X cleavage buffer (20 mM HEPES, pH 7.5, 150 mM KCl, 5% glycerol, 0.5 mM DTT), 5 mM MgCl_2_, 30 nM crRNA, and 25 nM FnoCas12a protein. The mixture was incubated at 37 °C for 5 min, followed by incubation on ice for 5 min. Subsequently, 100 ng of plasmid DNA containing the 5′-TTTA-3′ PAM and target sequence (Fig. 3*A*) was added to the mixture, and the reaction was incubated at 37 °C for various durations as needed. Reactions were terminated at different time points by adding an equal volume of 2X stop dye (final 1X concentration: 50 mM EDTA, 1% SDS, 10% glycerol, 0.04% orange G). Products were resolved on a 1% agarose gel and post-stained using ethidium bromide (0.5 μg/ml) and imaged using a Bio-Rad GelDoc.

The intensities of DNA bands corresponding to supercoiled precursor [P], nicked [N], and linear [L] forms were quantified using ImageJ 1.54g. Background contributions were corrected by baseline adjustment. Normalized values for each DNA form were calculated as follows:

Equation 1: Normalized fraction of nicked DNA.

(1) $$Normalized\;Frac\;\left[N\right]=\frac{\left[\left(\frac{I_{N}}{I_{N}+I_{L}+I_{SC}}\right)-{\left(\frac{I_{N}}{I_{N}+I_{L}+I_{SC}}\right)}_{C}\right]}{{\left(\frac{I_{SC}}{I_{N}+I_{L}+I_{SC}}\right)}_{C}}$$

Equation 2: Normalized fraction of linear DNA.

(2) $$Normalized\;Frac\;\left[L\right]=\frac{\left[\left(\frac{I_{L}}{I_{N}+I_{L}+I_{SC}}\right)-{\left(\frac{I_{L}}{I_{N}+I_{L}+I_{SC}}\right)}_{C}\right]}{{\left(\frac{I_{SC}}{I_{N}+I_{L}+I_{SC}}\right)}_{C}}$$

Equation 3: Normalized fraction of supercoiled precursor DNA.

(3) NormalizedFrac[P]=1−(Norm.Frac[N]+Norm.Frac[L])

Here, subscript 'C' represents values measured from enzyme-free control lanes. Negative values arising from trace amounts of nicked DNA in the control were set to zero. The overall cleavage fraction was calculated as:

Equation 4: Fraction of DNA cleaved.

(4) Totalcleavage[TC]=Norm.Frac[N]+Norm.Frac[L]

Experimental variation was quantified using standard deviation (SD), defined as:

Equation 5: Standard deviation, where x̄ is each replication value, x is the mean, and n is the number of replications.

(5) $$SD=\sqrt{\frac{\Sigma {\left(x-\bar{x}\right)}^{2}}{\left(n-1\right)}}$$

The rate constants and active fraction were determined by fitting the time-course data to a single-exponential rise model in which the supercoiled precursor (P) is converted to the linear product (L).

Equation 6: Single-exponential rise model.

(6) $$L\left(t\right)=A\cdot \left(1-e^{\;{-k}_{obs}.\;t}\right)$$

where L(t) represents the fraction of linearized substrate at time t, A is the active fraction, and *k*_*obs*_ is the observed rate constant.

### Mismatch cleavage assay

For the plasmid mismatch DNA cleavage assays, each Cas12a variant was diluted to a desired concentration using a 1X cleavage buffer consisting of 20 mM HEPES, pH 7.5, 150 mM KCl, 2 mM EDTA, 1 mM TCEP. All reactions were 10 μL in total volume and consisted of 25 nM protein, 30 nM crRNA, 100 ng DNA, and 5 mM MgCl_2_, and 1X cleavage buffer. FnoCas12a variants were preincubated with crRNA in reaction buffer at 37 °C for 5 min before addition of the plasmid DNA substrate. To initiate the reaction, 100 ng of mismatch plasmid DNA was added last and incubated at 37 °C. For this experiment, 20 mismatched plasmids (MM1-MM20) (Fig. 3*A*) were analyzed alongside the matched plasmid. All mismatched plasmids contained the same PAM sequence as the matched plasmid. The reaction was stopped after 60 min by the addition of a 2X stop dye (final 1X concentration: 50 mM EDTA, 1% SDS, 10% glycerol, 0.04% orange G). All reactions were subsequently run on a 1.1% agarose gel and bands were visualized with ethidium bromide (0.5 μg/mL) staining. Band intensities were then quantified using ImageJ 1.54g.

Within-protein substrate discrimination (R_1_ ratio) was calculated as the fold change in the matched (MCH) substrate cleavage relative to mismatched (MM). For each MM1–MM20 substrate, R_1_ was defined as the ratio of MCH cleavage to MM cleavage within the same protein. Cleavage fraction was calculated as.

Equation 7:

(7) $$R_{1}=\frac{{Normalized\;Frac\;\left[L\right]}_{MCH}}{{Normalized\;Frac\;\left[L\right]}_{MM}}$$

Where linear products are normalized using Equation 2. An R_1_ value of one indicates equal linearization of MCH and MM substrates, R_1_ > 1 indicates preferential linearization of the MCH substrate over the MM substrate, and 0 ≤ R_1_ < 1 indicates greater linearization of the MM substrate than the MCH substrate.

Variant-to-wild-type discrimination (R_2_ ratio) was calculated to compare substrate discrimination of each variant relative to FnoCas12a^WT^. R_2_ was defined as.

Equation 8:

(8) $$R_{2}=\frac{{\left(R_{1}\right)}_{variant}}{{\left(R_{1}\right)}_{WT}}$$

An R_2_ value of 1 indicates discrimination equivalent to FnoCas12a^WT^, R_2_ > 1 indicates improved mismatch discrimination relative to FnoCas12a^WT^, and 0 ≤ R_2_ < 1 indicates reduced mismatch discrimination relative to FnoCas12a^WT^.

### FAM-labeled DNA cleavage assay

The FAM-labeled DNA cleavage assay was performed using both matched and mismatch-16 linear oligo DNAs with the 5′-TTTA-3′ PAM (Table S10). For each substrate, two sets of oligo DNAs were used for the assays: one with a 5′ 6-FAM (Fluorescein) label on NTS and no label on the TS, and a second set with a 5′ 6-FAM label on the TS and no label on the NTS. Both sets of DNAs were annealed to form a 53-base pair DNA substrate using a 1X annealing buffer (30 mM HEPES pH 7.5 and 10 mM Potassium Acetate, KC_2_H_3_O_2_). For the reaction, 250 nM crRNA and 250 nM FnoCas12a were mixed in 1X cleavage buffer (20 mM Tris pH 7.5, 100 mM KCl, 5% (v/v) glycerol, and 10 mM MgCl_2_) and approximately 10 nM of annealed DNA was used to initiate the reaction. The reaction was stopped at various time points with a 1X stop dye containing 10 mM EDTA, 47% Formamide, and 1% SDS. The samples were resolved with a 16% denaturing polyacrylamide gel containing 8 M urea. The gel was then visualized using fluorescence with a 490 nm excitation using a Bio-Rad ChemiDoc MP CCD Imaging System. A fluorescent ssDNA ladder was prepared by mixing 5′ 6-FAM (Fluorescein) labeled DNA oligonucleotides of 53, 40, 29, 25, 19, and 15 nucleotides. The oligos were combined to a final concentration of 10 nM per oligo and used as a size reference for denaturing polyacrylamide gel. The 53-nt oligo corresponds to the full-length, uncleaved NTS or TS strand. For NTS cleavage, products range from 25 to 29-nt based on trimming and for TS cleavage the product is 21-nt long.

### Trans-cleavage assays

*Trans* cleavage assays were conducted using a 20-nt ssDNA activator complementary to the crRNA guide region. The substrate used was M13mp18 single-stranded DNA (NEB). FnoCas12a protein, crRNA, and the activator ssDNA were preincubated at concentrations of 25 nM, 30 nM, and 25 nM, respectively, in 1X cleavage buffer supplemented with 5 mM MgCl_2_ at 37 °C for 5 min. After incubation, the mixture was cooled on ice for 5 min, followed by the addition of 100 ng (∼4.4 nM) of M13mp18 single-stranded DNA. The reaction was then incubated at 37 °C. Reactions were stopped by adding an equal volume of 2X stop dye (final 1X concentration: 50 mM EDTA, 1% SDS, 10% glycerol, 0.04% orange G) and resolved on 1% agarose gels. Ethidium bromide (0.5 μg/mL) staining post the gel running was used for visualization, and band intensities were quantified using ImageJ 1.54g. To measure the loss of M13mp18 DNA, the intensity of the circular (I_Cr_) band was determined for both the control lane (I_Cr_ control) and the reaction lane (I_Cr_ sample). The fraction of circular DNA remaining in each lane was then calculated.

Equation 9: Relative Circular DNA Intensity.

(9) $$I_{Cr}=\left(\frac{I_{Cr}\;sample}{I_{Cr}\;control}\right)$$

The data were fit to a one-phase exponential decay model.

Equation 10:

(10) $$I_{Cr}\;\left(t\right)=Ae^{-k\text{obs}\;t}+\left(1-A\right)$$

where *I*_*Cr*_ (t) is the fraction of circular DNA remaining at time t. A represents the active fraction that undergoes cleavage, while (1 − A) represents the resistant fraction that remains unchanged. The rate at which the cleavable fraction decays is given by the observed rate constant, *k*_*obs*_.

### Alkaline agarose denaturing gel

Alkaline agarose denaturing gel electrophoresis was performed using 1% agarose gels (9). Agarose was dissolved in distilled water by heating, cooled to approximately 55 °C, and mixed with 0.1 volume of 10X alkaline buffer to obtain a final 1X alkaline buffer composition of 50 mM NaOH and 1 mM EDTA in the gel. Gels were cast, allowed to solidify, and presoaked in 1X alkaline buffer for 30 min before sample loading. Reactions were stopped using stop dye with a final 1X composition of 3% Ficoll, 50 mM EDTA, 1% SDS, and 0.04% xylene cyanol. NaOH was added to the samples and DNA ladder to a final concentration of 50 mM, followed by incubation for 5 min at room temperature and heat denaturation at 70 °C for 3 min. Samples were immediately cooled on ice and loaded onto the presoaked alkaline agarose gel. Electrophoresis was performed at 3 V/cm at 4 °C for 7 h. After electrophoresis, gels were neutralized in 1 M Tris-HCl, pH 7.6, and 1.5 M NaCl for 1 h, rinsed in water, stained with SYBR Green and visualized using a Bio-Rad ChemiDoc imaging system.

### RNA-independent DNA cleavage assay

RNA-independent DNA cleavage assays were conducted using M13mp18 single-stranded DNA (NEB) as the substrate in the presence of Mn^2+^ ions. FnoCas12a protein was prepared in 1X cleavage buffer containing MnCl_2_ at final concentration of 10 mM. The reaction mixture included 100 ng (∼4.4 nM) of M13mp18 single-stranded DNA incubated with FnoCas12a at 37 °C for a designated time period. Reactions were terminated by adding an equal volume of 2X stop dye (final 1X concentration: 50 mM EDTA, 1% SDS, 10% glycerol, 0.04% orange G), followed by separation of reaction products on 1% agarose gels. Visualization and quantification of DNA cleavage products were performed using ethidium bromide (0.5 μg/ml) staining and ImageJ 1.54g, respectively. The rate calculations were performed using the same single-exponential fitting procedure described above for *trans* cleavage.

### Computational modeling

We used structures of FnoCas12a transition state (PDB ID: 6GTG (24)) to construct the models for FnoCas12a^WT^, FnoCas12a^R918A^, FnoCas12a^R918K^, FnoCas12a^R921A^, FnoCas12a^R921K^, FnoCas12a^R918K/R921K^ for computational studies. Since the NTS-DNA is not fully resolved in the FnoCas12a active-pocket, we superimposed the available full AsCas12a structure (PDB ID: 8SFO (25)) onto FnoCas12a and used the DNA and Mg^2+^ ion coordinates from the AsCas12a model to complete the structure. The DNA sequences were then modified to match the FnoCas12a ternary complex structure (PDB ID: 6GTG). Protonation states were assigned by PROPKA (48, 49). Systems were solvated in TIP3P (50) water using a nearly cubic simulation box with at least 12 Å of solvent beyond the protein/crRNA/DNA complex in all directions, neutralized with Na^+^ counterions, and prepared with divalent metals at the catalytic site. Each system was heated to 300 K and equilibrated through sequential NVT and NPT stages (1 ns NVT heating followed by NPT equilibration at 1 bar, continued until the system density reached ∼1.01 g/cm^3^). Each system underwent 1 μs NVT simulation. From the resulting trajectories, the last 250 ns of the production step were concatenated and analyzed using Principal Component Analysis (PCA), totaling 1.5 μs after including all the systems. Similarly, for the Solvent-Accessible Surface Area (SASA) analysis, only the last 250 ns of each trajectory were used. Both PCA and SASA analyses were performed using CPPTRAJ (51). Simulations were performed using AMBER20 (52) employing AMBER/ff14SB (53), OL15 (54), ff99bsc0+χOL3 (55), and TIP3P (50) parameters for protein, DNA, RNA, and water, respectively. Additional computational details are provided in the Supplementary Information (Section S1).

## Data availability

All data supporting the findings, including uncropped gel images used for the activity assays, are included in the Supporting Information. Additional data for molecular dynamics simulations, including input files and simulation trajectories, are available upon request from Dr Yihan Shao (yihanshao@brandeis.edu).

## Supporting information

This article contains supporting information (9, 24, 25, 44, 48, 49, 50, 51, 52, 53, 54, 55, 56).

## Conflict of interest

The authors declare that they have no conflicts of interest with the contents of this article.
