# Supplementary material for: Active-site arginines differentially control Cas12a DNA cleavage and specificity
Source: J Biol Chem. 2026 Sep 16;302(11):113560. doi: 10.1016/j.jbc.2026.113560 (PMC13691569; doi:10.1016/j.jbc.2026.113560)
Supplement: Supplementary file 1 [file mmc1.docx]

**Supporting Information**

**Active-site arginines differentially control Cas12a DNA cleavage and specificity**

Saadi Rostami^1^, Alberto M. dos Santos^2^, Richard S. Van^2^, Kole Long^1^, Yihan Shao^2^, Rakhi Rajan^1*^

1- Department of Chemistry and Biochemistry, Price Family Foundation Institute of Structural Biology, Stephenson Life Sciences Research Center, The University of Oklahoma, Norman, Oklahoma, USA.

2- Department of Chemistry, Brandeis University, Waltham, MA 02453, USA.

Correspondence: [r-rajan@ou.edu](mailto:r-rajan@ou.edu)

***Running title:* RuvC arginines regulate Cas12a’s DNA cleavage**

**Table of Contents**

- Section S1. Computational modeling
- Figure S1. Sequence alignment of representative Cas12a orthologs
- Figure S2. Progressive remodeling of the RuvC active pocket during R-loop extension is accompanied by lid folding and repositioning of R909 in AsCas12a
- Figure S3. Proposed positioning of the non-target strand (NTS) within the RuvC active pocket of FnoCas12a
- Figure S4. Purification of FnoCas12a variants
- Figure S5. Kinetic analysis of linear product formation during cis cleavage by FnoCas12a variants
- Figure S6. Analysis of NTS and TS DNA cleavage patterns by FnoCas12a^WT^ and variants
- Figure S7. Initial rates of FAM-labeled DNA cleavage by FnoCas12a^WT^ and variants
- Figure S8. Native agarose gel analysis corresponding to the alkaline agarose gels shown in Figures 5A and 6A
- Figure S9. Pairwise 2D root mean square deviation (2D RMSD) of the simulation trajectories of FnoCas12a^WT^ and variants
- Figure S10. Principal component analysis (PCA) of crRNA–DNA complexes of FnoCas12a^WT^ and variants during MD simulations
- Figure S11. Residue flexibility analysis showing localized conformational changes outside the catalytic pocket in FnoCas12a^WT^ and variants
- Figure S12. Root mean square fluctuation (RMSF) analysis of nucleic acids in FnoCas12a^WT^ and variants during MD simulations
- Supplementary movie legends
- Table S1. Time-course *cis* cleavage of matched (MCH) plasmid DNA by FnoCas12a^WT^ and variants
- Table S2. Observed rate constants and fold-change reduction in *cis* cleavage activity for FnoCas12a^WT^ and variants
- Table S3. Measured values of nicked, linearized, and total DNA cleavage of mismatched substrates by FnoCas12a^WT^ and variants
- Table S4. Analysis of DNA mismatch discrimination potential by FnoCas12a^WT^ and variants
- Table S5. Analysis of NTS and TS cleavage efficiencies of FnoCas12a^WT^ and variants
- Table S6. Assessment of *trans*-cleavage efficiencies of FnoCas12a^WT^ and variants
- Table S7. Assessment of RNA-independent (R-I) DNA cleavage efficiencies of FnoCas12a^WT^ and variants
- Table S8. Observed rate constants and fold-change reduction in RNA-independent (R-I) DNA cleavage activity of FnoCas12a^WT^ and variants
- Table S9. Primers used in the study
- Table S10. Oligonucleotide DNAs used in the study
- Figure S13. Gel images showing three replicates of time-course *cis*-cleavage assays of FnoCas12a^WT^ and variants using MCH plasmid as the substrate
- Figure S14. Uncropped gel images of endpoint cleavage assays with FnoCas12a^WT^ and variants using MCH and MM1-MM20 substrates
- Figure S15. Uncropped gel images of three replicates of time-course *cis*-cleavage assays using FAM-labeled TS and NTS DNA oligonucleotides for MCH and MM16 substrates
- Figure S16. Replicate gel images for time-course *trans*-cleavage assays using a single-stranded activator and M13mp18 single-stranded DNA as the substrate
- Figure S17. Replicate gel images for time-course RNA-independent (R-I) DNA cleavage assays using M13mp18 single-stranded DNA as the substrate
- Figure S18. Uncropped alkaline and native agarose gel images showing *trans* and R-I cleavage for all variants
- Figure S19. Uncropped gels corresponding to Figure S6

Section S1. Computational modeling

Here, we present details of our molecular dynamics (MD) simulation study focused on investigating how substitutions at R918 and R921 alter the local conformational landscape of the RuvC active site of Cas12a protein. The pre-catalytic stage of *Francisella novicida* Cas12a (FnoCas12a^WT^) was obtained from the cryo-EM structure available under PDB ID: 6GTG (24), using DNA and Mg^2+^ coordinates from PDB ID: 8SFO (25). The nucleic acids were then modified to match the sequence present in 6GTG. Protonation states of titratable residues were predicted using PROPKA (48, 49). Each initial structure was solvated in a rectangular water box extending 12 Å from the protein/crRNA/DNA complex and neutralized with Na⁺ counterions. Systems were gradually heated from 0 K to 300 K via Langevin dynamics (friction coefficient: 5 ps⁻¹), followed by 1 ns of equilibration. Pressure was maintained at 1 atm using the Berendsen barostat under periodic boundary conditions until the system density reached ~1.01 g/cm3. All bonds containing hydrogen atoms were constrained using the SHAKE algorithm (51). Finally, a 1 μs production MD run was performed under NVT ensemble conditions and a time step of 1 fs. Simulations were performed using the AMBER20 (52) employing AMBER/ff14SB (53), OL15 (55), ff99bsc0+χOL3 (54), and TIP3P (50) parameters for protein, DNA, RNA, and water, respectively. Analyses were performed using CPPTRAJ (56).

In this study, we focused on the pre-catalytic stage. Analysis of the 2D-RMSD showed that all systems reached equilibrium after 250 ns of the 1 μs production MD simulations (Fig. S9). Therefore, the final 750 ns of each trajectory were used for subsequent structural analyses, ensuring that the reported distance, Solvent-Accessible Surface Area (SASA), Principal Component Analysis (PCA), and related measurements reflect equilibrated dynamics.

As shown in the 2D-RMSD (Fig. S9) and RMSF analyses (Fig. S11; Fig. S12), the substitutions did not cause global changes in the overall Cas12a structure. In order to evaluate local changes at the catalytic site, two distances were monitored. The first is the distance between the Cβ atom of residue 918 and the Cδ atom of Glu1028 (Distance #1) (Fig. 7A) and the second one is the distance between the Cβ atom of residue 921 and the phosphate group of 19^th^ nucleotide, from PAM, in the non-target strand (NTS) (Distance #2). The 19^th^ nucleotide was selected because its phosphate group interacts with R921 in the FnoCas12a^WT^ model and it is adjacent to the reported cut site at 18^th^ nucleotide (Fig. 7A; Fig. S3). These two measurements were selected to probe the coupling of residue 918 to the downstream helix element linked to RuvC-lid and the NTS positioning near residue 921. Upon closer inspection of those two key distances using two-dimensional distance analysis, variants showed distinct distributions of key distances within the catalytic site (Fig. 7B). This indicates that substitutions at these arginine positions alter the local arrangement of residue 918, the downstream helix, RuvC-lid, NTS and residue 921 near the catalytic site (Fig. 7B). FnoCas12a^WT^ and FnoCas12a^R921K^ exhibit closely related interactions, consistent with preservation of a productive state when a positive charge is retained at position 921 (Fig. 7B). In contrast, FnoCas12a^R921A^ shows a significant displacement in the NTS distance, supporting the idea that residue 921 contributes directly to positioning the NTS phosphate backbone near the catalytic site. FnoCas12a^R918K^ displays an intermediate behavior when compared to FnoCas12a^R921K^, whereas FnoCas12a^R918A^ shifts more strongly away from FnoCas12a^WT^ state in both distance coordinates, with a broader disruption of both coupling between the downstream helix/RuvC-lid and NTS substrate positioning (Fig. 7B).

To further investigate how these changes affect the catalytic site, we performed SASA analysis of the active-site region. The SASA calculation included the catalytic residues (D917, E1006, and D1255), nucleotides (15^th^ to 19^th^) from NTS, and both catalytic Mg²⁺ ions (Fig. 7C). Therefore, the observed shifts likely reflect coordinated changes in active-site architecture rather than isolated side-chain motions and can reveal the effects of amino acid substitutions on remodeling the local exposure to water molecules observed during the MD simulations. The distributions are narrowest for the more compact conformational states and broader for the more perturbed systems, with FnoCas12a^R921A^ showing the most solvent-exposed ensemble and FnoCas12a^R918A^ also displaying increased exposure when compared to FnoCas12a^WT^ (Fig. 7D). Variants with lysine substitution (FnoCas12a^R918K^, FnoCas12a^R921K^ and FnoCas12A^R918K/R921K^) show SASA distributions closest to that of FnoCas12a^WT^ (Fig. 7D). These observations suggest that removal of the native arginine side chains destabilizes the interactions that normally organize the active pocket and its surrounding nucleic acid contacts.

The PCA analysis allows reduction of the dimensionality of the MD trajectories by diagonalizing the covariance matrix of Cα atom coordinates, transforming correlated atomic motions into a set of orthogonal vectors (PCs) that capture collective movements of the protein in Cartesian space (44). In PCA, the eigenvalues reflect the RMSD of Cα atoms, with PC1 and PC2 representing the dominant motions across MD simulation. To explore the relationship between the different conformational states, PCA represented in two dimensions was obtained from probability distributions of both PC1 and PC2 for the concatenated MD trajectory comprising FnoCas12a^WT^ and variants FnoCas12a^R918A^, FnoCas12a^R918K^, FnoCas12a^R921A^, FnoCas12a^R921K^, and FnoCas12a^R918K/R921K^. The CPPTRAJ module available in the Amber20 package was used to analyze the coordinates of the Cα, C, N, O, P, C?', O?', and Mg^2+^ atoms over the last 750 ns of MD simulations to generate the principal components (PC1 and PC2) of the region surrounding the catalytic site (Fig. S10A). The results show that FnoCas12a^WT^ and each variant populate separable conformational minima (Fig. S10B). Along the first principal component (PC1) from right to left (Fig. S10B), a progression is observed in the order WT → R921K → R918K / R918K-R921K → R918A / R921A, delineating an order consistent with the activity observed in the experimental cleavage capability (Table S1). Along PC1, all substitutions appear to be associated with downstream helix being less helical and with a slight opening of the catalytic pocket (Fig. S10C; Movie S2). This indicates that the variants can generate distinguishable catalytic site dynamics over the MD simulation trajectory, supporting the observation that R918 and R921 make non-equivalent contributions to active-site organization (Fig. S10B).

**Figure S1. Sequence alignment of representative Cas12a orthologs.** Multiple sequence alignment of 91 different Cas12a orthologs including widely used *Acidaminococcus* sp. Cas12a (AsCas12a, green horizontal box), *Lachnospiraceae bacterium* Cas12a (LbCas12a, orange horizontal box), *Francisella novicida* Cas12a (FnoCas12a, purple horizontal box), and *Moraxella bovoculi* Cas12a (MbCas12a, gray horizontal box) orthologs. Conserved residues are highlighted in yellow. Red and black vertical boxes indicate arginine residues selected for substitution in this study. Cyan and blue vertical boxes indicate tyrosine and glutamic acid (Y1024 and E1028 in FnoCas12a), respectively, in the downstream helix (DH) that interact with R918 (of FnoCas12a) in different conformations of Cas12a. Full-length Cas12a protein sequences were aligned, and only the regions of interest are shown in the figure. Dots indicate sequence regions that are not displayed. Yellow bar graphs represent the percentage conservation at each position in the consensus sequence. "X" indicates positions without a strongly conserved amino acid, whereas "-" indicates a gap introduced during sequence alignment. Sequence labels were retained from the source database annotation. The dataset includes both well-characterized bacterial species and sequences derived from candidate taxa or metagenome-associated assemblies.

**Figure S2. Progressive remodeling of the RuvC active pocket during R-loop extension is accompanied by lid folding and repositioning of R909 in AsCas12a. (A-I)** Structural snapshots of AsCas12a cryo-EM complexes representing increasing R-loop lengths and distinct active-site conformations (PDB IDs and the corresponding R-loop lengths or active site status shown in each panel). The RuvC active pocket (salmon) is shown with the RuvC-lid region (green) and downstream helix (DH; cyan) highlighted separately to visualize conformational changes during DNA loading. The lid undergoes a gradual transition from a disordered loop state to an ordered α-helical conformation as R-loop formation progresses. This transition is accompanied by repositioning of residue R909 (equivalent of R918 in FnoCas12a) from the catalytic center towards the downstream helix. Distances shown in yellow indicate the distance between R909 and catalytic residue E993 (equivalent of E1006 in FnoCas12a) within the RuvC active site, whereas distances shown in black indicate the distance between R909 and E1016 (equivalent of E1028 in FnoCas12a) located on the downstream helix. Early intermediates show R909 positioned near the catalytic residue, while folding of the RuvC-lid correlates with increased separation from E993 and formation of interactions with Y1012 (equivalent of Y1024 in FnoCas12a, panel D) and E1016 from the downstream helix. Catalytic residues are labeled with red asterisks. In panel F, catalytic Mg^2+^ ions (lime) are shown. TS, target strand (blue); NTS, non-target strand (pale blue); BH, bridge helix (magenta); DH, downstream helix (cyan); crRNA (orange).

**Figure S3. Proposed positioning of the non-target strand (NTS) within the RuvC active pocket of FnoCas12a.** Structural model showing the positioning of the NTS DNA (turquoise) in the RuvC catalytic pocket of FnoCas12a after energy minimization with molecular dynamics simulation. The protein conformation was taken from the FnoCas12a cryo-EM structure (PDB ID: 6GTG (24)), whereas the NTS and Mg^2+^ conformations were derived from the corresponding regions of the AsCas12a structure (PDB ID: 8SFO (25)). DNA sequence was modified to match the missing DNA sequence in 6GTG. Mg²⁺ ions are shown as green spheres. Residues shown in gray indicate FnoCas12a residues predicted to participate in NTS stabilization based on structural correspondence to residues previously reported to interact with the NTS in AsCas12a (Fig. 1E). RuvC domain (salmon), Nuc domain (beige), RuvC-lid (green), bridge helix (magenta), and downstream helix/DH (cyan).

**Figure S4. Purification of FnoCas12a variants.** Representative SDS-PAGE analysis of 300 ng purified FnoCas12a proteins including FnoCas12a^WT^, FnoCas12a^R918K^, FnoCas12a^R918A^, FnoCas12a^R921K^, FnoCas12a^R921A^, and FnoCas12a^R918K/R921K^ (RRKK). For each protein variant, two independent protein preparations (Prep 1 and Prep 2) are shown. In this study, among the three replicates performed for each experiment, one replicate was conducted using protein Prep 1 and two replicates were conducted using protein Prep 2. Protein samples were visualized by Coomassie staining. The expected molecular weight of FnoCas12a is approximately 152 kDa. All variants show comparable purity.

**Figure S5. Kinetic analysis of linear product formation during *cis* cleavage by FnoCas12a variants. (A-F)** Accumulation of linearized cleavage product over time for FnoCas12a^WT^ and variants (FnoCas12a^R918K^, FnoCas12a^R918A^, FnoCas12a^R921K^, FnoCas12a^R921A^, and FnoCas12a^R918K/R921K^ (RRKK)) for *cis* cleavage. Data points (individual circles) represent the mean, and standard deviation (SD) from the three replicates is shown for each data point. Data points were fitted in MATLAB using single-exponential rise model, L(t) = A⋅(1−e^−^*^k^_obs_^.t^*), Equation 6, where A is active fraction and *k_obs_* is the observed rate constant. Solid lines represent the best-fit curves. Reported *k_obs_* and mean squared error (MSE) values are shown for each variant. MSE is reported as an indicator of goodness of fit and lower values indicate a closer fit.

**Figure S6. Analysis of NTS and TS DNA cleavage patterns by FnoCas12a^WT^ and variants. (A)** Schematic representation of the expected FAM-labeled cleavage products for MCH and MM16 substrates, showing NTS and TS strands and the positions of the labeled fragments. Cut sites between 18^th^ and 19^th^ nucleotides of NTS and between 21^st^ and 22^nd^ nucleotides of TS will create a 29-mer and a 21-mer FAM-labeled products, respectively. **(B)** Denaturing gel shows trimmed products generated by FnoCas12a^WT^ and the indicated variants for NTS cleavage (a 25-mer product corresponding to cleavage between 14^th^ and 15^th^ nucleotides) and the 21-mer product for TS cleavage, which is same for FnoCas12a^WT^ and variants. FnoCas12a^WT^ and single lysine substituents (FnoCas12a^R918K^ and FnoCas12a^R921K^) primarily produce one-sized product. In contrast, FnoCas12a^R918A^ and FnoCas12a^R918K/R921K^ (RRKK) exhibit slower trimming of NTS as indicated by intermediate products on the gel. **(C)** Denaturing gel showing products for FAM-labeled TS-MCH (left) and FAM-labeled NTS-MCH (right) cleavage by FnoCas12a^WT^ at 25 °C. Control lanes contain substrate only. Even at lower temperatures, FnoCas12a^WT^ produces the trimmed product band (25-mer) and no intermediate longer band (29-mer) as observed in the variants~~.~~

**Figure S7. Initial rates of FAM-labeled DNA cleavage by FnoCas12a^WT^ and variants.** Percent cleavage of FAM-labeled non-target strand (NTS) and target strand (TS) substrates for MCH and MM16 sequences over the initial time course. Initial rates were estimated from the slope of the linear region between 0 and 2.5 (as % cleavage min^-1^). Data points represent mean ± SD from three replications. The table summarizes the calculated slopes (as %/min) for each protein–substrate combination.

**Figure S8. Native agarose gel analysis corresponding to alkaline agarose gels shown in Figures 5A and 6A.**

(A) *Trans*-cleavage assay analyzed on a 1% native agarose gel. M13mp18 circular ssDNA was incubated with FnoCas12a–crRNA variants in the presence of ssDNA activator for 60 min. The control lane contains M13mp18 ssDNA with FnoCas12a–crRNA but without ssDNA activator. This native gel shows the mobility pattern of the DNA species present in the endpoint alkaline agarose gel shown in Figure 5A.

(B) RNA-independent DNA cleavage assay analyzed on a 1% native agarose gel. M13mp18 circular ssDNA was incubated with FnoCas12a variants in the absence of crRNA for 15 min. The control lane contains M13mp18 ssDNA without enzyme. This native gel shows the mobility pattern of the DNA species present in the endpoint alkaline agarose gel shown in Figure 6A.

C indicates intact circular M13mp18 ssDNA substrate, and D indicates degraded ssDNA products. A 1 kbp (kilo base pair) DNA ladder was used for molecular weight markers that are shown on the left of each gel.

**Figure S9. Pairwise 2D root mean square deviation (2D RMSD) of the simulation trajectories of FnoCas12a^WT^ and variants.** Each panel represents a different complex: **(A)** FnoCas12a^WT^, **(B)** FnoCas12a^R918K^, **(C)** FnoCas12a^R918A^, **(D)** FnoCas12a^R921K^, **(E)** FnoCas12a^R921A^, and **(F)** FnoCas12a^R918K/R921K^ (RRKK). Each colored point represents the RMSD between the frame conformation on the X-axis and the frame conformation on the Y-axis. The analysis reveals that all systems are moving into a single stable conformation at the end of the simulation.

**Figure S10. Principal component analysis (PCA) of crRNA-DNA complexes of FnoCas12a^WT^ and variants during MD simulations. (A)** Structural representation of the region selected for the PCA analysis. **(B)** Two-dimensional PCA plot of the conformational space sampled over the combined last 250 ns of the MD trajectories. Colors indicate the systems: FnoCas12a^WT^, dark olive; FnoCas12a^R918K^, red; FnoCas12a^R918A^, green; FnoCas12a^R921K^, blue; FnoCas12a^R921A^, orange and FnoCas12A^R918K/R921K^ (RRKK), purple. Essential motion obtained from PCA analysis for PC1 (47.1%) is shown in **(C)**, and that for PC2 (34.6%) is shown in **(D)**. Movies illustrating the dominant motions along PC1 (Movie S2) and PC2 (Movie S3) are provided to show these conformational changes.

**Figure S11. Residue flexibility analysis showing localized conformational changes outside the catalytic pocket in FnoCas12a^WT^ and variants.** **(A)** Root mean square fluctuation (RMSF) profiles calculated from MD trajectories for FnoCas12a^WT^ and variants FnoCas12a^R918K^, FnoCas12a^R918A^, FnoCas12a^R921K^, FnoCas12a^R921A^, and FnoCas12a^R918K/R921K^ (RRKK). RMSF values are plotted as a function of residue number. **(B)** Residues showing RMSF values differing by ≥ 2 Å relative to the corresponding residue in FnoCas12a^WT^ are highlighted. Colors indicate the variant in which the fluctuation was observed and correspond to the color scheme used in panel **A**. **(C-G)** Structural mapping of residues identified in panel **B** onto the FnoCas12a structure for FnoCas12a^R918K^ **(C)**, FnoCas12a^R918A^ **(D)**, FnoCas12a^R921K^ **(E)**, FnoCas12a^R921A^ **(F)**, and FnoCas12a^R918K/R921K^ (RRKK) (**G)**. Most residues exhibiting fluctuations are located on solvent-exposed regions of the protein surface and are primarily associated with loop regions. These residues are generally positioned distal to the RuvC active pocket. For FnoCas12a^R921K^, residues appearing near the center of the bilobe are located on the backside of the presented view and the closest residue within this region is located 33.6 Å from the Mg²⁺ ions in the active pocket.

**Figure S12. Root mean square fluctuation (RMSF) analysis of nucleic acids in FnoCas12a^WT^ and variants during MD simulations. (A-C)** RMSF profiles calculated from the last 250 ns of the MD trajectories for target strand DNA (TS-DNA) **(A)**, non-target strand DNA (NTS-DNA) **(B)**, and crRNA **(C)** in FnoCas12a^WT^ and variants FnoCas12a^R918K^, FnoCas12a^R918A^, FnoCas12a^R921K^, FnoCas12a^R921A^, and FnoCas12a^R918K/R921K^ (RRKK). Colors indicate the corresponding protein variant as shown in the label for Figure S12A. The X-axis shows the nucleotide sequence for each nucleic acid component. Nucleotides with RMSF values differing by ≥ 2 Å relative to wild type (WT) are highlighted with hollow circles. **(D)** Structural mapping of nucleotides identified by the RMSF analysis. Nucleic acids are colored as crRNA (orange), TS (gray), and NTS (turquoise). Red triangles indicate the expected cut sites: 18^th^ nucleotide for NTS within the protein active site and 23^rd^ nucleotide for TS in the RNA-DNA hybrid schematic. For TS-DNA, higher fluctuations are mainly observed at the 5′ and 3′ termini, which have limited protein contacts. For NTS-DNA, increased fluctuations occur at the termini and central target region, consistent with greater flexibility of the single-stranded region. Notably, NTS nucleotides 10-12 in FnoCas12a^R918K/R921K^ show increased RMSF relative to FnoCas12a^WT^, and these are positioned outside the RuvC active pocket and remain solvent exposed. In FnoCas12a^R921A^, increased fluctuation is also observed for NTS nucleotide 5.

**Supplementary movie legends**

**Movie S1.** The movie displays the folding of RuvC-lid from a loop to an α-helical form in AsCas12a shown in Figure S2.

**Movie S2.** The movie depicts the dominant motions along PC1 from the Principal Component Analysis, corresponding to the PCA analysis in Figure S10C.

**Movie S3.** The movie depicts the dominant motions along PC2 from the Principal Component Analysis, corresponding to the PCA analysis in Figure S10D.

| **FnoCas12a^WT^** | | | | **FnoCas12a^R918K^** | | | |
| --- | --- | --- | --- | --- | --- | --- | --- |
| T (min) | SC (%) | N (%) | L (%) | T (min) | SC (%) | N (%) | L (%) |
| 0 | 100 ± 0 | 0 ± 0 | 0 ± 0 | 0 | 100 ± 0 | 0 ± 0 | 0 ± 0 |
| 0.25 | 14 ± 14 | 28 ± 19 | 59 ± 33 | 0.25 | 65 ± 29 | 22 ± 14 | 13 ± 17 |
| 0.5 | 4 ± 0 | 18 ± 28 | 78 ± 28 | 0.5 | 43 ± 26 | 33 ± 10 | 24 ± 24 |
| 1 | 3 ± 2 | 4 ± 10 | 93 ± 10 | 1 | 24 ± 9 | 32 ± 12 | 44 ± 17 |
| 2.5 | 0 ± 0 | 0 ± 0 | 100 ± 0 | 2.5 | 19 ± 7 | 21 ± 14 | 59 ± 16 |
| 5 | 0 ± 0 | 0 ± 0 | 100 ± 0 | 5 | 18 ± 7 | 11 ± 9 | 71 ± 10 |
| 7.5 | 0 ± 0 | 0 ± 0 | 100 ± 0 | 7.5 | 16 ± 9 | 0 ± 2 | 84 ± 10 |
| 10 | 0 ± 0 | 0 ± 0 | 100 ± 0 | 10 | 13 ± 7 | 0 ± 0 | 87 ± 9 |
| 15 | 0 ± 0 | 0 ± 0 | 100 ± 0 | 15 | 12 ± 5 | 0 ± 0 | 88 ± 5 |
| 30 | 0 ± 0 | 0 ± 0 | 100 ± 0 | 30 | 9 ± 5 | 0 ± 0 | 91 ± 7 |
| 45 | 0 ± 0 | 0 ± 0 | 100 ± 0 | 45 | 6 ± 5 | 0 ± 0 | 94 ± 5 |
| 60 | 0 ± 0 | 0 ± 0 | 100 ± 0 | 60 | 4 ± 3 | 0 ± 0 | 96 ± 5 |
| **FnoCas12a^R918A^** | | | | **FnoCas12a^R921K^** | | | |
| T (min) | SC (%) | N (%) | L (%) | T (min) | SC (%) | N (%) | L (%) |
| 0 | 100 ± 0 | 0 ± 0 | 0 ± 0 | 0 | 100 ± 0 | 0 ± 0 | 0 ± 0 |
| 0.25 | 98 ± 2 | 2 ± 2 | 0 ± 0 | 0.25 | 41 ± 16 | 32 ± 12 | 27 ± 7 |
| 0.5 | 90 ± 7 | 9 ± 7 | 1 ± 0 | 0.5 | 33 ± 19 | 9 ± 5 | 58 ± 14 |
| 1 | 78 ± 7 | 14 ± 3 | 8 ± 2 | 1 | 29 ± 19 | 6 ± 2 | 66 ± 19 |
| 2.5 | 69 ± 3 | 21 ± 2 | 9 ± 2 | 2.5 | 25 ± 16 | 8 ± 2 | 67 ± 16 |
| 5 | 45 ± 3 | 37 ± 3 | 19 ± 2 | 5 | 24 ± 12 | 9 ± 2 | 67 ± 16 |
| 7.5 | 20 ± 2 | 42 ± 2 | 38 ± 2 | 7.5 | 22 ± 12 | 10 ± 3 | 68 ± 16 |
| 10 | 13 ± 2 | 41 ± 2 | 46 ± 2 | 10 | 23 ± 12 | 12 ± 2 | 65 ± 14 |
| 15 | 11 ± 3 | 35 ± 3 | 53 ± 3 | 15 | 21 ± 10 | 12 ± 2 | 67 ± 12 |
| 30 | 10 ± 3 | 32 ± 2 | 58 ± 3 | 30 | 20 ± 10 | 13 ± 2 | 68 ± 10 |
| 45 | 10 ± 5 | 23 ± 2 | 68 ± 5 | 45 | 18 ± 9 | 14 ± 0 | 69 ± 9 |
| 60 | 6 ± 3 | 13 ± 3 | 81 ± 7 | 60 | 18 ± 7 | 14 ± 2 | 67 ± 7 |
| **FnoCas12a^R921A^** | | | | **FnoCas12a^R918K/R921K^** | | | |
| T (min) | SC (%) | N (%) | L (%) | T (min) | SC (%) | N (%) | L (%) |
| 0 | 100 ± 0 | 0 ± 0 | 0 ± 0 | 0 | 100 ± 0 | 0 ± 0 | 0 ± 0 |
| 0.25 | 100 ± 0 | 0 ± 0 | 0 ± 0 | 0.25 | 95 ± 7 | 5 ± 7 | 0 ± 0 |
| 0.5 | 100 ± 0 | 0 ± 0 | 0 ± 0 | 0.5 | 86 ± 14 | 13 ± 12 | 1 ± 2 |
| 1 | 100 ± 0 | 0 ± 0 | 0 ± 0 | 1 | 67 ± 19 | 28 ± 12 | 6 ± 7 |
| 2.5 | 100 ± 0 | 0 ± 0 | 0 ± 0 | 2.5 | 48 ± 26 | 37 ± 7 | 16 ± 19 |
| 5 | 100 ± 2 | 0 ± 2 | 0 ± 2 | 5 | 23 ± 9 | 45 ± 5 | 33 ± 14 |
| 7.5 | 100 ± 0 | 0 ± 0 | 0 ± 0 | 7.5 | 11 ± 2 | 41 ± 5 | 49 ± 5 |
| 10 | 100 ± 0 | 0 ± 0 | 0 ± 0 | 10 | 9 ± 2 | 37 ± 5 | 55 ± 3 |
| 15 | 100 ± 0 | 0 ± 0 | 0 ± 0 | 15 | 8 ± 2 | 27 ± 3 | 65 ± 2 |
| 30 | 99 ± 2 | 0 ± 2 | 1 ± 2 | 30 | 8 ± 2 | 18 ± 5 | 74 ± 3 |
| 45 | 99 ± 0 | 0 ± 0 | 1 ± 2 | 45 | 7 ± 3 | 9 ± 3 | 85 ± 5 |
| 60 | 99 ± 0 | 0 ± 0 | 1 ± 2 | 60 | 6 ± 3 | 4 ± 2 | 90 ± 5 |

**Table S1. Time-course *cis* cleavage of matched (MCH) plasmid DNA by FnoCas12a^WT^ and variants.** *Cis* cleavage of the matched plasmid substrate was monitored over time for FnoCas12a^WT^, FnoCas12a^R918K^, FnoCas12a^R918A^, FnoCas12a^R921K^, FnoCas12a^R921A^, and the double variant FnoCas12a^R918K/R921K^. The percentages of supercoiled (SC), nicked (N), and linearized (L) plasmid species are reported at each time point. Values represent the mean ± SD of three replications.

| **Variant** | **k_obs_ ± SD (min^-1^)** | **MSE** | **Fold Change Reduction** |
| --- | --- | --- | --- |
| FnoCas12a^WT^ | 3.24 ± 0.13 | 0.000021 | N.A. |
| FnoCas12a^R918K^ | 0.49 ± 0.43 | 0.000263 | 6.6 |
| FnoCas12a^R918A^ | 0.08 ± 0.01 | 0.000244 | 40.5 |
| FnoCas12a^R921K^ | 2.83 ± 0.91 | 0.000096 | 1.1 |
| FnoCas12a^R921A^ | n.p. | n.p. | N.A. |
| FnoCas12a^R918K/R921K^ | 0.10 ± 0.03 | 0.000098 | 32.4 |

**Table S2. Observed rate constants and fold-change reduction in *cis* cleavage activity for FnoCas12a^WT^ and variants.** Observed rate constants (k_obs_) were obtained by fitting time-course cleavage data to a single-exponential growth model for FnoCas12a^WT^ and the indicated variants. Values are reported as mean ± SD. The mean squared error (MSE) reflects the quality of each fit and lower values indicate a closer fit. Fold-change reduction was calculated by dividing the k_obs_ value of wild-type by that of each variant. N.A., not applicable. n.p., not provided.

| **Substrate** | **FnoCas12a^WT^** | | | **FnoCas12a^R918K^** | | |
| --- | --- | --- | --- | --- | --- | --- |
|  | **Nicked (%)** | **Linear (%)** | **Total Cleavage (%)** | **Nicked (%)** | **Linear (%)** | **Total Cleavage (%)** |
| MCH | 4 ± 0 | 82 ± 7 | 86 ± 7 | 1 ± 0 | 90 ± 2 | 91 ± 2 |
| MM1 | 2 ± 2 | 62 ± 5 | 65 ± 5 | 4 ± 3 | 91 ± 3 | 95 ± 2 |
| MM2 | 2 ± 2 | 59 ± 9 | 60 ± 10 | 2 ± 2 | 85 ± 3 | 87 ± 3 |
| MM3 | 4 ± 0 | 67 ± 7 | 71 ± 7 | 4 ± 0 | 90 ± 3 | 94 ± 5 |
| MM4 | 3 ± 2 | 63 ± 9 | 66 ± 7 | 3 ± 2 | 83 ± 9 | 86 ± 7 |
| MM5 | 3 ± 2 | 62 ± 3 | 65 ± 3 | 3 ± 2 | 84 ± 5 | 88 ± 3 |
| MM6 | 3 ± 2 | 80 ± 10 | 83 ± 9 | 3 ± 0 | 94 ± 0 | 97 ± 0 |
| MM7 | 4 ± 3 | 85 ± 7 | 89 ± 3 | 6 ± 2 | 90 ± 3 | 96 ± 3 |
| MM8 | 6 ± 2 | 89 ± 2 | 95 ± 2 | 4 ± 3 | 96 ± 3 | 99 ± 0 |
| MM9 | 15 ± 7 | 73 ± 10 | 88 ± 5 | 19 ± 3 | 77 ± 3 | 96 ± 0 |
| MM10 | 12 ± 3 | 77 ± 10 | 89 ± 7 | 10 ± 2 | 86 ± 2 | 96 ± 2 |
| MM11 | 7 ± 3 | 85 ± 5 | 93 ± 2 | 4 ± 2 | 92 ± 2 | 96 ± 2 |
| MM12 | 39 ± 3 | 59 ± 3 | 98 ± 0 | 26 ± 5 | 72 ± 5 | 98 ± 2 |
| MM13 | 26 ± 7 | 73 ± 7 | 98 ± 2 | 38 ± 3 | 60 ± 3 | 99 ± 2 |
| MM14 | 39 ± 2 | 59 ± 2 | 98 ± 2 | 48 ± 3 | 50 ± 3 | 98 ± 2 |
| MM15 | 38 ± 3 | 57 ± 3 | 95 ± 2 | 38 ± 5 | 58 ± 5 | 96 ± 2 |
| MM16 | 28 ± 0 | 67 ± 2 | 95 ± 2 | 79 ± 0 | 20 ± 0 | 98 ± 0 |
| MM17 | 17 ± 3 | 76 ± 5 | 93 ± 3 | 34 ± 3 | 61 ± 3 | 95 ± 2 |
| MM18 | 11 ± 2 | 81 ± 3 | 91 ± 2 | 12 ± 3 | 83 ± 3 | 95 ± 0 |
| MM19 | 1 ± 3 | 85 ± 14 | 87 ± 10 | 4 ± 3 | 85 ± 3 | 90 ± 2 |
| MM20 | 7 ± 7 | 74 ± 7 | 81 ± 5 | 0 ± 2 | 85 ± 7 | 85 ± 3 |
| **Substrate** | **FnoCas12a^R918A^** | | | **FnoCas12a^R921K^** | | |
|  | **Nicked (%)** | **Linear (%)** | **Total Cleavage (%)** | **Nicked (%)** | **Linear (%)** | **Total Cleavage (%)** |
| MCH | 25 ± 2 | 53 ± 3 | 78 ± 2 | 9 ± 3 | 82 ± 3 | 91 ± 0 |
| MM1 | 28 ± 3 | 53 ± 0 | 81 ± 3 | 11 ± 3 | 83 ± 3 | 94 ± 0 |
| MM2 | 20 ± 5 | 43 ± 7 | 63 ± 10 | 6 ± 2 | 71 ± 5 | 77 ± 7 |
| MM3 | 24 ± 2 | 53 ± 5 | 77 ± 7 | 13 ± 3 | 75 ± 9 | 88 ± 7 |
| MM4 | 21 ± 3 | 51 ± 7 | 71 ± 10 | 9 ± 2 | 64 ± 19 | 74 ± 19 |
| MM5 | 23 ± 2 | 41 ± 3 | 65 ± 5 | 12 ± 0 | 63 ± 7 | 76 ± 5 |
| MM6 | 33 ± 3 | 37 ± 3 | 70 ± 7 | 10 ± 0 | 75 ± 3 | 85 ± 3 |
| MM7 | 35 ± 3 | 43 ± 2 | 77 ± 5 | 15 ± 2 | 73 ± 2 | 88 ± 0 |
| MM8 | 26 ± 7 | 63 ± 10 | 89 ± 2 | 12 ± 3 | 87 ± 3 | 98 ± 2 |
| MM9 | 60 ± 3 | 15 ± 2 | 76 ± 5 | 29 ± 9 | 67 ± 12 | 96 ± 3 |
| MM10 | 55 ± 3 | 27 ± 2 | 82 ± 3 | 28 ± 5 | 69 ± 5 | 96 ± 3 |
| MM11 | 42 ± 2 | 39 ± 3 | 81 ± 5 | 18 ± 5 | 79 ± 5 | 98 ± 2 |
| MM12 | 82 ± 2 | 15 ± 2 | 97 ± 2 | 60 ± 3 | 39 ± 3 | 98 ± 2 |
| MM13 | 87 ± 2 | 7 ± 2 | 94 ± 2 | 61 ± 2 | 39 ± 2 | 100 ± 0 |
| MM14 | 92 ± 3 | 3 ± 2 | 95 ± 2 | 73 ± 2 | 26 ± 2 | 99 ± 0 |
| MM15 | 87 ± 2 | 5 ± 2 | 92 ± 2 | 64 ± 3 | 33 ± 2 | 98 ± 2 |
| MM16 | 95 ± 2 | 1 ± 0 | 95 ± 2 | 78 ± 2 | 21 ± 2 | 99 ± 0 |
| MM17 | 85 ± 2 | 6 ± 2 | 91 ± 2 | 38 ± 5 | 58 ± 5 | 96 ± 2 |
| MM18 | 62 ± 2 | 29 ± 3 | 92 ± 2 | 30 ± 0 | 66 ± 2 | 96 ± 0 |
| MM19 | 1 ± 0 | 77 ± 3 | 78 ± 2 | 1 ± 0 | 91 ± 2 | 92 ± 2 |
| MM20 | 0 ± 0 | 28 ± 3 | 28 ± 3 | 2 ± 2 | 80 ± 2 | 82 ± 2 |
| **Substrate** | **FnoCas12a^R918K/R921K^** | | |  | | |
|  | **Nicked (%)** | **Linear (%)** | **Total Cleavage (%)** |  |  |  |
| MCH | 18 ± 3 | 63 ± 2 | 81 ± 3 |  |  |  |
| MM1 | 15 ± 2 | 74 ± 5 | 89 ± 3 |  |  |  |
| MM2 | 9 ± 5 | 61 ± 9 | 70 ± 10 |  |  |  |
| MM3 | 14 ± 2 | 59 ± 14 | 73 ± 17 |  |  |  |
| MM4 | 12 ± 2 | 53 ± 12 | 65 ± 12 |  |  |  |
| MM5 | 13 ± 2 | 47 ± 7 | 60 ± 7 |  |  |  |
| MM6 | 29 ± 2 | 53 ± 5 | 82 ± 7 |  |  |  |
| MM7 | 29 ± 3 | 40 ± 2 | 70 ± 3 |  |  |  |
| MM8 | 23 ± 3 | 76 ± 3 | 98 ± 2 |  |  |  |
| MM9 | 64 ± 0 | 17 ± 0 | 81 ± 2 |  |  |  |
| MM10 | 55 ± 5 | 30 ± 2 | 85 ± 5 |  |  |  |
| MM11 | 31 ± 14 | 39 ± 21 | 70 ± 36 |  |  |  |
| MM12 | 84 ± 7 | 10 ± 2 | 94 ± 5 |  |  |  |
| MM13 | 90 ± 3 | 5 ± 2 | 95 ± 3 |  |  |  |
| MM14 | 95 ± 2 | 3 ± 2 | 98 ± 0 |  |  |  |
| MM15 | 90 ± 2 | 5 ± 2 | 95 ± 2 |  |  |  |
| MM16 | 95 ± 2 | 1 ± 0 | 95 ± 2 |  |  |  |
| MM17 | 89 ± 0 | 7 ± 0 | 96 ± 2 |  |  |  |
| MM18 | 67 ± 7 | 26 ± 5 | 93 ± 3 |  |  |  |
| MM19 | 2 ± 2 | 72 ± 5 | 74 ± 5 |  |  |  |
| MM20 | 1 ± 0 | 37 ± 5 | 38 ± 3 |  |  |  |

**Table S3. Tables showing the measured values of nicked, linear, and total DNA cleavage of mismatched (MM) substrates by FnoCas12a^WT^ and variants.** Each table summarizes cleavage activity against matched (MCH) and MM1-20 DNA substrates. Cleavage outcomes are categorized into nicked and linear forms, with total cleavage calculated as the sum of both. Values are reported as mean ± SD from three replicates.

**A**

|  | **FnoCas12a** | | | | |
| --- | --- | --- | --- | --- | --- |
| **R_1_ ratio**  **(within-protein discrimination)** | **WT** | **R918K** | **R918A** | **R921K** | **R918K/R921K** |
| MCH/MM1 | 1.3 | 1.0 | 1.0 | 1.0 | 0.9 |
| MCH/MM2 | 1.4 | 1.1 | 1.2 | 1.2 | 1.0 |
| MCH/MM3 | 1.2 | 1.0 | 1.0 | 1.1 | 1.1 |
| MCH/MM4 | 1.3 | 1.1 | 1.0 | 1.3 | 1.2 |
| MCH/MM5 | 1.3 | 1.1 | 1.3 | 1.3 | 1.3 |
| MCH/MM6 | 1.0 | 1.0 | 1.4 | 1.1 | 1.2 |
| MCH/MM7 | 1.0 | 1.0 | 1.2 | 1.1 | 1.6 |
| MCH/MM8 | 0.9 | 0.9 | 0.8 | 0.9 | 0.8 |
| MCH/MM9 | 1.1 | 1.2 | 3.5 | 1.2 | 3.7 |
| MCH/MM10 | 1.1 | 1.0 | 2.0 | 1.2 | 2.1 |
| MCH/MM11 | 1.0 | 1.0 | 1.4 | 1.0 | 1.6 |
| MCH/MM12 | 1.4 | 1.3 | 3.6 | 2.1 | 6.5 |
| MCH/MM13 | 1.1 | 1.5 | 8.0 | 2.1 | 12.7 |
| MCH/MM14 | 1.4 | 1.8 | 16.1 | 3.1 | 18.2 |
| MCH/MM15 | 1.4 | 1.6 | 10.7 | 2.4 | 12.9 |
| MCH/MM16 | 1.2 | 4.6 | 53.0 | 3.8 | 63.0 |
| MCH/MM17 | 1.1 | 1.5 | 8.9 | 1.4 | 9.3 |
| MCH/MM18 | 1.0 | 1.1 | 1.8 | 1.2 | 2.4 |
| MCH/MM19 | 1.0 | 1.1 | 0.7 | 0.9 | 0.9 |
| MCH/MM20 | 1.1 | 1.1 | 1.9 | 1.0 | 1.7 |

**B**

|  | **FnoCas12a** | | | |
| --- | --- | --- | --- | --- |
| **R_2_ ratio**  **(variant-to-wild-type discrimination)** | **R918K** | **R918A** | **R921K** | **R918K/R921K** |
| (MCH_variant_/MM1_variant_)/(MCH_WT_/MM1_WT_) | 0.7 | 0.8 | 0.7 | 0.6 |
| (MCH_variant_/MM2_variant_)/(MCH_WT_/MM2_WT_) | 0.8 | 0.9 | 0.8 | 0.7 |
| (MCH_variant_/MM3_variant_)/(MCH_WT_/MM3_WT_) | 0.8 | 0.8 | 0.9 | 0.9 |
| (MCH_variant_/MM4_variant_)/(MCH_WT_/MM4_WT_) | 0.8 | 0.8 | 1.0 | 0.9 |
| (MCH_variant_/MM5_variant_)/(MCH_WT_/MM5_WT_) | 0.8 | 1.0 | 1.0 | 1.0 |
| (MCH_variant_/MM6_variant_)/(MCH_WT_/MM6_WT_) | 0.9 | 1.4 | 1.1 | 1.2 |
| (MCH_variant_/MM7_variant_)/(MCH_WT_/MM7_WT_) | 1.0 | 1.3 | 1.2 | 1.6 |
| (MCH_variant_/MM8_variant_)/(MCH_WT_/MM8_WT_) | 1.0 | 0.9 | 1.0 | 0.9 |
| (MCH_variant_/MM9_variant_)/(MCH_WT_/MM9_WT_) | 1.0 | 3.1 | 1.1 | 3.3 |
| (MCH_variant_/MM10_variant_)/(MCH_WT_/MM10_WT_) | 1.0 | 1.8 | 1.1 | 2.0 |
| (MCH_variant_/MM11_variant_)/(MCH_WT_/MM11_WT_) | 1.0 | 1.4 | 1.1 | 1.7 |
| (MCH_variant_/MM12_variant_)/(MCH_WT_/MM12_WT_) | 0.9 | 2.6 | 1.5 | 4.6 |
| (MCH_variant_/MM13_variant_)/(MCH_WT_/MM13_WT_) | 1.3 | 7.1 | 1.9 | 11.2 |
| (MCH_variant_/MM14_variant_)/(MCH_WT_/MM14_WT_) | 1.3 | 11.5 | 2.2 | 13.0 |
| (MCH_variant_/MM15_variant_)/(MCH_WT_/MM15_WT_) | 1.1 | 7.4 | 1.7 | 9.0 |
| (MCH_variant_/MM16_variant_)/(MCH_WT_/MM16_WT_) | 3.7 | 43.1 | 3.1 | 51.4 |
| (MCH_variant_/MM17_variant_)/(MCH_WT_/MM17_WT_) | 1.4 | 8.3 | 1.3 | 8.6 |
| (MCH_variant_/MM18_variant_)/(MCH_WT_/MM18_WT_) | 1.1 | 1.8 | 1.2 | 2.4 |
| (MCH_variant_/MM19_variant_)/(MCH_WT_/MM19_WT_) | 1.1 | 0.7 | 0.9 | 0.9 |
| (MCH_variant_/MM20_variant_)/(MCH_WT_/MM20_WT_) | 1.0 | 1.7 | 0.9 | 1.6 |

**Table S4. Analysis of DNA mismatch discrimination potential by FnoCas12a^WT^ and variants. (A)** Fold change in mismatch (MM1-20) plasmid substrate linearization by FnoCas12a^WT^ and variants relative to MCH linearization to find within-protein discrimination (R_1_ ratio). Each value represents the ratio: (MCH _linearization_) / (MM _linearization_) for that specific protein. An R_1_ value of 1 indicates equal linearization of the MCH and MM substrates. R_1_ > 1 indicates preferential linearization of the MCH substrate over the MM substrate, with larger values indicating stronger mismatch discrimination. A value of 0 ≤ R_1_ < 1 indicates greater linearization of the MM substrate than the MCH substrate. **(B)** Fold change for variant-to-wild-type discrimination (R_2_ ratio) by FnoCas12a^WT^ and variants. This table shows the fold change in linearization of MCH over MM1-20 plasmids by each variant relative to the corresponding ratio of FnoCas12a^WT^. Each value represents the ratio: (MCH _variant_ / MM _variant_) / (MCH _WT_ / MM _WT_). An R_2_ value of 1 indicates discrimination equivalent to FnoCas12a^WT^. R_2_ > 1 indicates improved mismatch discrimination relative to FnoCas12a^WT^, whereas 0 ≤ R_2_ < 1 indicates reduced mismatch discrimination relative to FnoCas12a^WT^.

.

| **A** | **FnoCas12a^WT^** | | | |
| --- | --- | --- | --- | --- |
|  | MCH Cleavage (%) | | MM16 Cleavage (%) | |
| Time (min) | FAM-NTS | FAM-TS | FAM-NTS | FAM-TS |
| 0 | 0 ± 0 | 0 ± 0 | 0 ± 0 | 0 ± 0 |
| 2.5 | 71 ± 3 | 67 ± 5 | 37 ± 17 | 24 ± 3 |
| 10 | 77 ± 3 | 75 ± 2 | 49 ± 14 | 41 ± 2 |
| 30 | 83 ± 5 | 84 ± 2 | 68 ± 10 | 60 ± 9 |
| 60 | 84 ± 5 | 86 ± 5 | 74 ± 2 | 68 ± 7 |

| **B** | **FnoCas12a^R918A^** | | | |
| --- | --- | --- | --- | --- |
|  | MCH Cleavage (%) | | MM16 Cleavage (%) | |
| Time (min) | FAM-NTS | FAM-TS | FAM-NTS | FAM-TS |
| 0 | 0 ± 0 | 0 ± 0 | 0 ± 0 | 0 ± 0 |
| 2.5 | 36 ± 9 | 26 ± 16 | 5 ± 2 | 2 ± 2 |
| 10 | 56 ± 12 | 45 ± 7 | 13 ± 12 | 6 ± 14 |
| 30 | 65 ± 5 | 55 ± 10 | 17 ± 14 | 6 ± 7 |
| 60 | 77 ± 2 | 64 ± 10 | 20 ± 10 | 11 ± 12 |

| **C** | **FnoCas12a^R918K/R921K^** | | | |
| --- | --- | --- | --- | --- |
|  | MCH Cleavage (%) | | MM16 Cleavage (%) | |
| Time (min) | FAM-NTS | FAM-TS | FAM-NTS | FAM-TS |
| 0 | 0 ± 0 | 0 ± 0 | 0 ± 0 | 0 ± 0 |
| 2.5 | 38 ± 14 | 29 ± 10 | 4 ± 2 | 3 ± 5 |
| 10 | 46 ± 23 | 37 ± 9 | 9 ± 19 | 8 ± 14 |
| 30 | 58 ± 19 | 54 ± 3 | 12 ± 12 | 10 ± 14 |
| 60 | 73 ± 19 | 72 ± 14 | 18 ± 5 | 13 ± 12 |

**Table S5. Analysis of NTS and TS cleavage efficiencies of FnoCas12a^WT^ and variants.** Quantified *cis*-cleavage of FAM-labeled MCH- and MM16-DNA substrates by FnoCas12a^WT^ **(A)**, FnoCas12a^R918A^ **(B)**, and FnoCas12a^R918K/R921K^ **(C).** Depending on the position of the FAM label on either the non-target strand (FAM-NTS) or the target strand (FAM-TS), the percentage of cleavage corresponds to strand-specific DNA cleavage. Percent cleavage was calculated as 100% minus the percent remaining substrate for each replication. Values are reported as mean ± SD from three replicates.

| **Time (min)** | **FnoCas12a (% substrate remaining)** | | | | | |
| --- | --- | --- | --- | --- | --- | --- |
|  | **WT** | **R918K** | **R918A** | **R921K** | **R921A** | **R918K/R921K** |
| 0 | 100 ± 0 | 100 ± 0 | 100 ± 0 | 100 ± 0 | 100 ± 0 | 100 ± 0 |
| 0.25 | 82 ± 19 | 98 ± 5 | 100 ± 3 | 100 ± 2 | 98 ± 9 | 100 ± 2 |
| 0.5 | 23 ± 19 | 95 ± 9 | 99 ± 5 | 99 ± 8 | 97 ± 9 | 98 ± 3 |
| 1 | 1 ± 2 | 93 ± 9 | 100 ± 5 | 99 ± 6 | 93 ± 9 | 96 ± 5 |
| 2.5 | 0 ± 0 | 93 ± 9 | 99 ± 9 | 99 ± 2 | 97 ± 9 | 97 ± 7 |
| 5 | 0 ± 0 | 96 ± 9 | 99 ± 7 | 100 ± 1 | 100 ± 10 | 98 ± 7 |
| 7.5 | 0 ± 0 | 93 ± 9 | 99 ± 7 | 98 ± 3 | 98 ± 7 | 96 ± 5 |
| 10 | 0 ± 0 | 98 ± 12 | 98 ± 9 | 99 ± 3 | 97 ± 12 | 96 ± 5 |
| 20 | 0 ± 0 | 97 ± 12 | 94 ± 10 | 100 ± 3 | 93 ± 12 | 98 ± 5 |
| 30 | 0 ± 0 | 94 ± 12 | 95 ± 10 | 100 ± 2 | 92 ± 14 | 100 ± 2 |
| 45 | 0 ± 0 | 98 ± 7 | 96 ± 10 | 99 ± 1 | 90 ± 10 | 98 ± 3 |
| 60 | 0 ± 0 | 95 ± 3 | 97 ± 7 | 99 ± 1 | 91 ± 12 | 97 ± 7 |

**Table S6. Assessment of *trans*-cleavage efficiencies of FnoCas12a^WT^ and variants.** Time-course *trans* cleavage assays with single-stranded plasmid DNA substrate is shown. FnoCas12a was activated with a 20-nt ssDNA activator that is fully complementary to the crRNA guide region. *Trans* cleavage activity was measured as the percentage of remaining substrate in each lane compared to that in the control lane at the indicated time points. Values are reported as mean ± SD from three replicates.

| **Time**  **(min)** | **FnoCas12a (% substrate remaining)** | | | | | |
| --- | --- | --- | --- | --- | --- | --- |
|  | **WT** | **R918K** | **R918A** | **R921K** | **R921A** | **R918K/R921K** |
| 0 | 100 ± 0 | 100 ± 0 | 100 ± 0 | 100 ± 0 | 100 ± 0 | 100 ± 0 |
| 0.25 | 70 ± 9 | 96 ± 3 | 100 ± 0 | 91 ± 12 | 93 ± 7 | 100 ± 2 |
| 0.5 | 19 ± 9 | 95 ± 3 | 97 ± 2 | 79 ± 16 | 84 ± 16 | 91 ± 2 |
| 1 | 12 ± 3 | 94 ± 9 | 94 ± 3 | 51 ± 16 | 85 ± 16 | 89 ± 5 |
| 2.5 | 11 ± 7 | 80 ± 16 | 95 ± 3 | 12 ± 5 | 83 ± 12 | 89 ± 7 |
| 5 | 7 ± 7 | 70 ± 12 | 80 ± 12 | 7 ± 2 | 83 ± 10 | 72 ± 3 |
| 7.5 | 6 ± 7 | 54 ± 17 | 70 ± 16 | 6 ± 3 | 75 ± 16 | 52 ± 3 |
| 10 | 2 ± 2 | 47 ± 14 | 61 ± 16 | 4 ± 2 | 73 ± 12 | 43 ± 5 |
| 20 | 0 ± 0 | 25 ± 10 | 41 ± 12 | 1 ± 0 | 69 ± 10 | 23 ± 5 |
| 30 | 0 ± 0 | 15 ± 2 | 22 ± 16 | 0 ± 0 | 48 ± 5 | 12 ± 3 |
| 45 | 0 ± 0 | 9 ± 2 | 13 ± 9 | 0 ± 0 | 37 ± 16 | 4 ± 2 |
| 60 | 0 ± 0 | 5 ± 2 | 9 ± 9 | 0 ± 0 | 33 ± 3 | 2 ± 0 |

**Table S7. Assessment of RNA-independent (R-I) DNA cleavage efficiencies of FnoCas12a^WT^ and variants.** Cleavage assays were performed in the absence of crRNA and in the presence of 10 mM Mn^2+^. Single-stranded plasmid DNA was used as the substrate. R-I cleavage activity was measured as the percentage of remaining substrate at the indicated time points. Values are reported as mean ± SD from three replicates.

| **Protein** | **k_obs_ ± SD (min^-1^)** | **MSE** | **Fold Change Reduction** |
| --- | --- | --- | --- |
| FnoCas12a^WT^ | 2.41 ± 0.23 | 0.000406 | N.A. |
| FnoCas12a^R918K^ | 0.08 ± 0.04 | 0.000061 | 30.1 |
| FnoCas12a^R918A^ | 0.05 ± 0.02 | 0.000074 | 48.2 |
| FnoCas12a^R921K^ | 0.66 ± 0.26 | 0.000163 | 3.6 |
| FnoCas12a^R921A^ | 0.05 ± 0.04 | 0.000420 | 48.2 |
| FnoCas12a^R918K/R921K^ | 0.08 ± 0.00 | 0.000122 | 30.1 |

**Table S8. Observed rate constants and fold-change reduction in RNA- independent (R-I) DNA cleavage activity of FnoCas12a^WT^ and variants.** Observed rate constants (k_obs_) were obtained by fitting time-course cleavage data to a single-exponential decay model for FnoCas12a^WT^ and the variants. Values are reported as mean ± SD. The mean squared error (MSE) reflects the quality of each fit and lower values indicate a closer fit. Fold-change reduction was calculated by dividing the k_obs_ value of FnoCas12a^WT^ by that of each variant. N.A.: not applicable.

| **Primers used** | **Construct** |
| --- | --- |
| FnoCas12a^WT^ is used from a previous study (9)  NCBI Reference Sequence: WP_003040289.1 | FnoCas12a in pET28m  [His8-3C-His6-MBPTEV- FnoCas12a] |
| FnoCas12A^R918K^-FWD:  CATATATTAAGTATAGATAAAGGTGAAAGACATTTAGC  FnoCas12A^R918K^-REV:  GCTAAATGTCTTTCACCTTTATCTATACTTAATATATG | FnoCas12A^R918K^ in  pET28m [His8-3CHis6-MBP-TEV-FnoCas12a] |
| FnoCas12A^R918A^-FWD:  CATATATTAAGTATAGATGCAGGTGAAAGACATTTAGC  FnoCas12A^R918A^-REV:  GCTAAATGTCTTTCACCTGCATCTATACTTAATATATG | FnoCas12A^R918A^ in  pET28m [His8-3CHis6-MBP-TEV-FnoCas12a] |
| FnoCas12A^R921K^-FWD:  GATAGAGGTGAAAAACATTTAGCTTACTATACTTTGG  FnoCas12A^R921K^-REV:  CCAAAGTATAGTAAGCTAAATGTTTTCACCTCTATC | FnoCas12A^R921K^ in  pET28m [His8-3CHis6-MBP-TEV-FnoCas12a] |
| FnoCas12A^R921A^-FWD:  GTATAGATAGAGGTGAAGCACATTTAGCTTACTATAC  FnoCas12A^R921A^-REV:  GTATAGTAAGCTAAATGTGCTTCACCTCTATCTATAC | FnoCas12A^R921A^ in  pET28m [His8-3CHis6-MBP-TEV-FnoCas12a] |
| FnoCas12A^R918K/R921K^-FWD:  ATTAAGTATAGATAAAGGTGAAAAACATTTAGCTTAC  FnoCas12A^R918K/R921K^-REV:  GTAAGCTAAATGTTTTTCACCTTTATCTATACTTAAT  Note: FnoCas12a^R918K^ was used as the template. | FnoCas12A^R918K/R921K^ in  pET28m [His8-3CHis6-MBP-TEV-FnoCas12a] |

**Table S9: Primers used in the study.** All sequences are listed in the 5' to 3' direction. The nucleotide substitutions are indicated by the underlined text.

| **DNA oligos** | **Purpose** |
| --- | --- |
| crRNA-template strand for matched plasmid and oligo assays: AGTGGCCTTATTAAATGACTTCTCATCTACAACAGTAGAAATTC*CCTATAGTGAGTCGTATTA*ATTTC  *T7 promoter* top strand:  GAAAT*TAATACGACTCACTATAGG*G | FnoCas12a- matched  DNA crRNA  transcription  oligos |
| 20-nt ssDNA activator:  GCCTTATTAAATGACTTCTC | Activation of FnoCas12a for *trans* cleavage initiation |
| FAM-labeled matched DNA oligo (TS):  GTCGTCAATTCTTTTAACAGTGGCCTTATTAAATGACTTCTC**TAAA**GGATCAT  FAM-labeled matched DNA oligo (NTS):  ATGATCC**TTTA**GAGAAGTCATTTAATAAGGCCACTGTTAAAAGAATTGACGAC  FAM-labeled MM16 DNA oligo (TS):  GTCGTCAATTCTTTTAACAGTGGCCT**C**ATTAAATGACTTCTC**TAAA**GGATCAT  FAM-labeled MM16 DNA oligo (NTS):  ATGATCC**TTTA**GAGAAGTCATTTAAT**G**AGGCCACTGTTAAAAGAATTGACGAC  Note: Oligo DNAs without FAM labeling had the same sequences as those reported above. PAM sequence is shown in bold. | FAM-labeled DNA cleavage Assay |

**Table S10: Oligo DNAs used in the study.** All sequences are listed in the 5′ to 3′ direction. The T7 promoter sequence is indicated by the italicized and underlined text, PAM sequences are shown in bold, and MM positions are indicated by bold and underlined nucleotides.

**A: FnoCas12a^WT^ – MCH Substrate**

**B: FnoCas12a^R918K^ – MCH Substrate**

**C: FnoCas12a^R918A^ – MCH Substrate**

**D: FnoCas12a^R921K^ – MCH Substrate**

**E: FnoCas12a^R921A^ – MCH Substrate**

**F: FnoCas12a^R918K/R921K^ – MCH Substrate**

**Figure S13. Gel images showing three replications for time-course *cis* cleavage assays of FnoCas12a^WT^ and variants using the MCH plasmid as the substrate. (A)** FnoCas12a^WT^ activity assay replicates with MCH substrate used for the quantification shown in Figure 2A. **(B)** FnoCas12a^R918K^ activity assay replicates with MCH substrate used for the quantification shown in Figure 2B. **(C)** FnoCas12a^R918A^ activity assay replicates with MCH substrate used for the quantification shown in Figure 2C. **(D)** FnoCas12a^R921K^ activity assay replicates with MCH substrate used for the quantification shown in Figure 2D. **(E)** FnoCas12a^R921A^ activity assay replicates with MCH substrate used for the quantification shown in Figure 2E. **(F)** FnoCas12a^R918K/R921K^ activity assay replicates with MCH substrate used for the quantification shown in Figure 2F. Assay was allowed to proceed for time points 15 sec, 30 sec, 1 min, 2.5 min, 5 min, 7.5 min, 10 min, 20 min, 30 min, 45 min, and 60 min. Gels outlined with a black frame indicates the replicate shown as the representative image in the main text (Figure 2).

**A. Lane order: MCH (Control, WT, R918K, R918A, R921K, RRKK), MM1 (WT, R918K, R918A, R921K, RRKK), MM2 (WT, R918K, R918A, R921K, RRKK).**

**B. Lane order: MM3 (WT, R918K, R918A, R921K, RRKK), MM4 (WT, R918K, R918A, R921K, RRKK), MM5 (WT, R918K, R918A, R921K, RRKK).**

**C. Lane order: MM6 (WT, R918K, R918A, R921K, RRKK), MM7 (WT, R918K, R918A, R921K, RRKK), MM8 (WT, R918K, R918A, R921K, RRKK).**

**D. Lane order: MM9 (WT, R918K, R918A, R921K, RRKK), MM10 (WT, R918K, R918A, R921K, RRKK), MM11 (WT, R918K, R918A, R921K, RRKK).**

**E. Lane order: MM12 (WT, R918K, R918A, R921K, RRKK), MM13 (WT, R918K, R918A, R921K, RRKK), MM14 (WT, R918K, R918A, R921K, RRKK).**

**F. Lane order: MM15 (WT, R918K, R918A, R921K, RRKK), MM16 (WT, R918K, R918A, R921K, RRKK), MM17 (WT, R918K, R918A, R921K, RRKK).**

**G. Lane order: MM18 (WT, R918K, R918A, R921K, RRKK), MM19 (WT, R918K, R918A, R921K, RRKK), MM20 (WT, R918K, R918A, R921K, RRKK).**

**Figure S14. Uncropped gel images of endpoint (60 min) cleavage assays with FnoCas12a^WT^ and variants using MCH and MM 1 to 20 substrates.** Three replications of each substrate are shown in the same row. These gels were used for quantification and correspond to the data presented in Figures 3A-E. [RRKK: FnoCas12a^R918K/R921K^].

**A: FnoCas12a^WT^ with FAM-labeled MCH DNA oligonucleotide substrate**

**B: FnoCas12a^R918A^ with FAM-labeled MCH DNA oligonucleotide substrate**

**C: FnoCas12a^R918K/R921K^ with FAM-labeled MCH DNA oligonucleotide**

**D: FnoCas12a^WT^ with FAM-labeled MM16 DNA oligonucleotide substrate**

**E: FnoCas12a^R918A^ with FAM-labeled MM16 DNA oligonucleotide substrate**

**F: FnoCas12a^R918K/R921K^ with FAM-labeled MM16 DNA oligonucleotide**

**Figure S15. Uncropped gel images of three replicates of time course *cis* cleavage assays of FAM-labeled target strand (TS) and non-target strand (NTS) DNA oligonucleotides for MCH and MM16 substrates by FnoCas12a^WT^ and variants. (A)** FnoCas12a^WT^ with FAM-labeled MCH DNA oligonucleotide substrate. These gels were used for quantification in Figure 4A. **(B)** FnoCas12a^R918A^ with FAM-labeled MCH DNA oligonucleotide substrate. These gels were used for quantification in Figure 4B. **(C)** FnoCas12a^R918K/R921K^ (RRKK) with FAM-labeled MCH DNA oligonucleotide substrate. These gels were used for quantification in Figure 4C. **(D)** FnoCas12a^WT^ with FAM-labeled MM16 DNA oligonucleotide substrate. These gels were used for quantification in Figure 4D. **(E)** FnoCas12a^R918A^ with FAM-labeled MM16 DNA oligonucleotide substrate. These gels were used for quantification in Figure 4E. **(F)** FnoCas12a^R918K/R921K^ (RRKK) with FAM-labeled MM16 DNA oligonucleotide substrate. These gels were used for quantification in Figure 4F. Assay was allowed to proceed for time points 2.5 min, 10 min, 30 min, and 60 min. Gels outlined with a black frame indicates the replicate shown as the representative image in the main text (Figure 4). The ladder shows oligonucleotides of 53, 40, 29, 25, 19, and 15 ssDNA nucleotides. Three replications for each combination is shown in one row. Note: The 60-min R918A-MCH-TS and R918A-MCH-NTS samples were inadvertently loaded in swapped positions; the lane labels indicate their correct sample identities.

**A: FnoCas12a^WT^ - M13mp18 single-stranded DNA Substrate**

**B: FnoCas12a^R918K^ - M13mp18 single-stranded DNA Substrate**

**C: FnoCas12a^R918A^ - M13mp18 single-stranded DNA Substrate**

**D: FnoCas12a^R921K^ - M13mp18 single-stranded DNA Substrate**

**E: FnoCas12a^R921A^ - M13mp18 single-stranded DNA Substrate**

**F: FnoCas12a^R918K/R921K^ - M13mp18 single-stranded DNA Substrate**

**Figure S16. Gel images of the replicates for the time course for *trans* cleavage assays of FnoCas12a^WT^ and variants using single strand activator and M13mp18 single-stranded DNA as substrate.** **(A)** FnoCas12a^WT^ activity assay replicates used for the quantification shown in Figure 5B. **(B)** FnoCas12a^R918K^ activity assay replicates used for the quantification shown in Figure 5C. **(C)** FnoCas12a^R918A^ activity assay replicates used for the quantification shown in Figure 5D. **(D)** FnoCas12a^R921K^ activity assay replicates used for the quantification shown in Figure 5E. **(E)** FnoCas12a^R921A^ activity assay replicates used for the quantification shown in Figure 5F. **(F)** FnoCas12a^R918K/R921K^ activity assay replicates used for the quantification shown in Figure 5G. Assay was allowed to proceed for time points 15 sec, 30 sec, 1 min, 2.5 min, 5 min, 7.5 min, 10 min, 20 min, 30 min, 45 min, and 60 min. Gels outlined with a black frame indicates the replicate shown as the representative image in the main figures. Three replications for each combination is shown in one row.

**A: FnoCas12a^WT^ - M13mp18 single-stranded DNA Substrate**

**B: FnoCas12a^R918K^ - M13mp18 single-stranded DNA Substrate**

**C: FnoCas12a^R918A^ - M13mp18 single-stranded DNA Substrate**

**D: FnoCas12a^R921K^ - M13mp18 single-stranded DNA Substrate**

**E: FnoCas12a^R921A^ - M13mp18 single-stranded DNA Substrate**

**F: FnoCas12a^R918K/R921K^ - M13mp18 single-stranded DNA Substrate**

**Figure S17. Gel images of the replicates for the time course for RNA-independent (R-I) DNA cleavage assays with FnoCas12a^WT^ and variants using M13mp18 single-stranded DNA as the substrate.** In this assay, no crRNA was used, and the reaction was initiated with 10 mM Mn²⁺ instead of Mg²⁺ **(A)** FnoCas12a^WT^ activity assay replicates used for the quantification shown in Figure 6B. **(B)** FnoCas12a^R918K^ activity assay replicates used for the quantification shown in Figure 6C. **(C)** FnoCas12a^R918A^ activity assay replicates used for the quantification shown in Figure 6D. **(D)** FnoCas12a^R921K^ activity assay replicates used for the quantification shown in Figure 6E. **(E)** FnoCas12a^R921A^ activity assay replicates used for the quantification shown in Figure 6F. **(F)** FnoCas12a^R918K/R921K^ activity assay replicates used for the quantification shown in Figure 6G. Assay was allowed to proceed for time points 15 sec, 30 sec, 1 min, 2.5 min, 5 min, 7.5 min, 10 min, 20 min, 30 min, 45 min, and 60 min. Gels outlined with a black frame indicates the replicate shown as the representative image in the main text (Figure 6). Three replications for each combination is shown in one row.

**Figure S18. Uncropped alkaline and native agarose gel images showing *trans* and R-I cleavage for all variants.** Panel A and B show the uncropped gel for the *trans* cleavage assay corresponding to Figures 5A and S8A, respectively. Panel C and D show the uncropped gel for the R-I DNA cleavage assay corresponding to Figure 6A and S8B, respectively. The last lane in each gel contains M13mp18 ssDNA treated with EcoRI as an attempt to linearize the ssDNA plasmid that has only one EcoRI site in the sequence. Despite having one cut-site in M13mp18ssDNA, the secondary structure of the ssDNA causes non-specific banding pattern. Since EcoRI treatment did not generate a clean, discrete linear product that can be used as a control, this lane was excluded from the cropped panels in the main figure. Note that with *trans* and R-I DNA cleavages, the product is degradation of ssDNA, and hence linear band production is not expected.

**Figure S19. Uncropped gels corresponding to Figure S6. (A)** Full gel image used for the cropped panels shown in Figure S6B. **(B)** Full gel image used for the cropped panels shown in Figure S6C.
